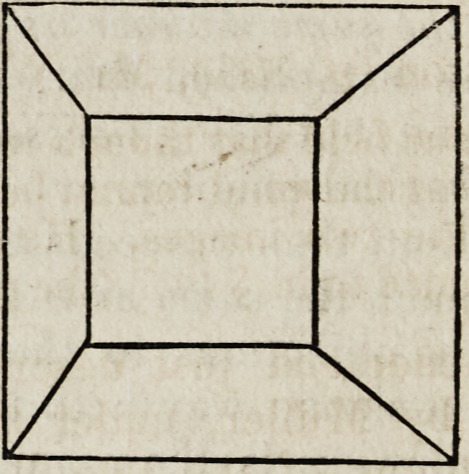# Elements of Ophthalmic Medicine and Surgery

**Published:** 1840-07

**Authors:** 


### BRITISH AND FOREIGN
### MEDICAL REVIEW,

FOR JULY, 1840.

### PART FIRST.

### Analytical and Critical Reviews.

### Art. I.

1. *Grundriss der gesammten Augenheilkunde.* Von Dr. August Andreæ. Erster Theil: *Allgemeine Augenheilkunde, mit drei Steindrucktafeln.* Zweiter Theil: *Specielle Augenheilkunde.—Magdeburg*, 1834-7. 8vo, pp. 123-559.

*Elements of Ophthalmic Medicine and Surgery.* By Dr. Augustus Andreæ. First Part, *containing the Generalities of the Subject.* Second Part, *containing the Specialities.—Magdeburg*, 1834-7.

2. *Dissertatio Inauguralis medico-literaria complectens Conspectum Historicum Scholæ Clinicæ Ophthalmiatricæ Viennensis, &c.* Auctore Anthonio Hardwiger.—*Viennæ*, 1836.

*An Historical View of the Ophthalmological Clinic of Vienna.* By Anthony Hardwiger.—*Vienna*, 1836.

3. *On Single and Correct Vision, by means of Double and Inverted Images on the Retinæ.* By W. P. Alison, m.d., f.r.s.e., Professor of the Institutes of Medicine in the University of Edinburgh. (*In the Transactions of the Royal Society of Edinburgh*, vol. xiii.)—*Edinburgh*, 1836.

4. *Contributions to the Physiology of Vision.* Part the First. *On some remarkable and hitherto unobserved Phenomena of Binocular Vision.* By Charles Wheatstone, f.r.s., Professor of Experimental Philosophy in King's College, London. (*In the Philosophical Transactions.* Part ii., 1838.)—*London*, 1838.

5. *Klinische Darstellungen der Krankheiten und Bildungsfehler des menschlichen Auges, der Augenlider und der Thränenwerkzeuge nach eigenen Beobachtungen und Untersuchungen.* Herausgegeben von Dr. Friedrich August von Ammon, Leibarzte Sr. Majestät des Königs von Sachsen, &c. Erster Theil, enthaltend: *Klinische Darstellungen der Krankheiten des menschlichen Auges. Hierzu drei hundert sieben und siebzig illuminirte Figuren auf drei und*

*zwanzig Tafeln. Folio.—Berlin,* 1838. Zweiter Theil, enthaltend: *Klinische Darstellungen der Krankheiten der Augenlider, der Augenhöhle und der Thränenwerkzeuge. Hierzu zwei hundert und zehn illuminirte Figuren auf zwölf Tafeln. Folio.—Berlin,* 1838.

*Clinical Illustrations of the Diseases and Malformations of the Human Eye, the Eyelids, and Lachrymal Organs, founded on the Author's own Observations and Researches.* By Dr. FREDERICK AUGUSTUS VON AMMON, Physician in Ordinary to the King of Saxony, &c. First Part, containing *Clinical Illustrations of the Diseases of the Human Eye. With Three Hundred and Seventy-seven coloured Figures in Twenty-three Plates. Folio.—Berlin,* 1838. Second Part, containing *Clinical Illustrations of the Diseases of the Eyelids, the Orbits, and Lachrymal Organs. With Two Hundred and Ten coloured Figures in Twelve Plates. Folio.—Berlin,* 1838.

6. *De Iritide. Commentatio ab illust. Societate medico-practica quæ Lutetiæ Parisiorum floret, &c., premio aureo publice ornata.* Scripsit FRID. AUG. AB AMMON, D.M., Potentissimi regis Saxoniæ *Archiater,* &c. *Cum tab. æn.* ii.—*Lipsiæ,* 1838.

*On Iritis. An Essay to which a gold medal was awarded by the Society of Practical Medicine of Paris.* By Dr. F. A. VON AMMON, Physician in Ordinary to the King of Saxony, &c. *With Two Copperplates.—Leipzig,* 1838.

7. *Guide Pratique pour l'Etude et le Traitement des Maladies des Yeux.* Par CH. J. F. CARRON DU VILLARDS, Docteur en Médecine et Chirurgie, Professeur d'Ophthalmologie à Paris, &c.—*Paris,* 1838. Deux tomes, 8vo.

*Practical Guide to the Study and Treatment of the Diseases of the Eyes.* By CH. J. F. CARRON DU VILLARDS, M. & C.D., Lecturer on Ophthalmology in Paris. *With Plates.* In two volumes, 8vo. Vol. I. pp. 556. Vol. II. pp. 644.

8. *Handbuch der Physiologie des Menschen.* Von Dr. JOHANNES MÜLLER, &c. Zweiten Bandes, Zweite Abtheilung: *Der speciellen Physiologie* Fünftes Buch: *Von den Sinnen.* I. Abschnitt, *Vom Gesichtssinn.*

*Elements of Physiology.* By J. MULLER, M.D., Professor of Anatomy and Physiology in the University of Berlin, &c. *Translated from the German with Notes,* by WILLIAM BALY, M.D., Graduate of the University of Berlin. Part V., containing *the Senses.* Section I. *of Vision.—London,* 1839.

9. *Cours d'Ophthalmologie, ou Traité complet des Maladies de l'Œil, professé publiquement à l'Ecole pratique de Médecine de Paris.* Par M. ROGNETTA, Docteur en Médecine et en Chirurgie, Professeur particulier de Pathologie externe, &c.—*Paris,* 1839. 8vo, pp. 468.

*Lectures on Ophthalmology, delivered publicly at the Practical School of Medicine of Paris; or a Complete Treatise on the Diseases of the Eye.* By M. ROGNETTA, M. & C.D., Lecturer on Surgery.—*Paris,* 1839.

10. *Handbuch der Augenheilkunde zum Gebrauche bei seinen Vorlesungen.* Von MAXIMILIAN JOSEPH CHELIUS, der Medicin und Chirurgie Doctor, ordentlichem öffentlichen Professor der Chirurgie und Augenheilkunde, Director der chirurgischen und Augenkranken-Klinik zu Heidelberg, &c. Zweiter Band, *Die organischen Krankheiten des Auges enthaltend.—Stuttgart,* 1839. 8vo, pp. 552.

*Manual of Ophthalmic Medicine and Surgery, for the use of his Pupils.* By Maximilian Joseph Chelius, m. & c.d., Professor of Surgery and Ophthalmology, and Director of the Surgical and Ophthalmic Clinic at Heidelberg, &c. Second Volume, *containing the Organic Diseases of the Eye.—Stuttgart,* 1839.

11. *Die sogenannte contagiöse oder ägyptische Augenentzündung. Eine Monographie.* Von Burkard Eble, Doctor der Medecin und Chirurgie, Bibliothekar der medicinisch-chirurgischen Josephsakademie zu Wien, &c. *Mit* 9 *colorirten Abbildungen.—Stuttgart,* 1839. 8vo, pp. 267.

*The so-called Contagious or Egyptian Ophthalmia. A Monography.* By Burkard Eble, m. & c.d., Librarian of the Josephine Academy of Vienna. *With Nine coloured Figures.—Stuttgart,* 1839.

12. *An Introductory Lecture on the Anatomy, Physiology, and Diseases of the Eye, delivered at the Birmingham Royal School of Medicine and Surgery, October* 4, 1839. By Richard Middlemore, Surgeon to the Birmingham Eye Infirmary, &c.—*London,* 1839. 8vo, pp. 30.

13. *A Practical Treatise on the Diseases of the Eye.* By William Mackenzie, m.d., Surgeon Oculist in Scotland in Ordinary to her Majesty, Lecturer on the Eye in the University of Glasgow, and one of the Surgeons to the Glasgow Eye Infirmary. *To which is prefixed an Anatomical Introduction, explanatory of a Horizontal Section of the Human Eyeball.* By Thomas Wharton Jones, Surgeon. Third Edition.—*London,* 1840. 8vo, pp. 923.

14. *Manuel Pratique des Maladies des Yeux, d'après les Leçons Cliniques de M. le Professeur Velpeau, Chirurgien de l'Hôpital de la Charité.* Par Gustave Jeanselme.—*Paris,* 1840. 18mo, pp. 676.

*A Practical Manual of the Diseases of the Eyes, composed from the Clinical Lectures of Professor Velpeau, Surgeon of the Hospital of La Charité.* By Gustavus Jeanselme.—*Paris,* 1840.

15. *Traité de Pathologie externe et de Médecine opératoire.* Par Aug. Vidal (de Cassis), Chirurgien de l'Hôpital de Lourcine, Professeur agrégé à la Faculté de Médecine de Paris, Professeur particulier de Pathologie externe et de Médecine opératoire.—*Paris,* 1840. Tome troisième. (*Maladies des Yeux.*) 8vo, pp. 588.

*A Treatise on Surgical Pathology and on Operative Surgery.* By Aug. Vidal (de Cassis), Surgeon of the Hospital of Lourcine, Lecturer on Surgery, &c.—*Paris,* 1840. Vol. III. (*Diseases of the Eye.*)

True as it is, that the diseases of the eye do not differ essentially from the diseases of any other part of the body, and that the general treatment of all must be regulated by the same principles, it is not to be denied that in the pathology and therapeutics of the eye there are specialities which in many cases so preponderate over the generalities as very much to obscure the analogy. The complete analogy in fact between the diseases of the eye and those of the rest of the body is not an *à priori* matter. It is only to be perfectly comprehended *à posteriori;* that is, when we are so far acquainted with both classes of diseases as to be able to distinguish what is fundamental, from what is accessory, and then excluding the latter, subject the former only to our comparison. Pursued in this manner the study of the diseases of the eye is found to illustrate, as well as to be illustrated by, those of other parts of the body. The points, however, in the pathology and therapeutics of the eye which demand special consideration are so numerous, so complicated, and often so much out of the way of general pathology and therapeutics, that, considering the importance of the sense of vision, a course of medical education cannot be considered complete unless ophthalmology has for some time especially engaged the student's attention. And here we would remark that to study ophthalmology effectively, an extensive and accurate knowledge of anatomy and physiology is the best preparation.

Ophthalmiatry took its rise in Egypt—in that country where diseases of the eye are endemic. The oculists of Egypt, indeed, were in early times in request among the other nations of the East, as appears from the story in Herodotus about Cyrus, King of Persia, sending to Amazis, king of Egypt, for the most expert oculist of his dominions. From Egypt the art of treating diseases of the eye was introduced into Greece. The Greeks being good observers, we find that, notwithstanding the want of accurate anatomical knowledge, which materially obstructed the study of the diseases of other organs, they were enabled, on account of the exposed situation of the eye and its transparency, to become great proficients in ophthalmiatry. Some idea of the extent of the knowledge of the diseases of the eye possessed by the Greeks may be had by considering that many of the names now in use have actually descended from them, and by casting a glance at the summary given by Celsus; for, though Celsus wrote at Rome, it is to be remembered his surgery was entirely that of the Greeks. As the Greeks received from the Egyptians their first instructions in the art of healing, so the Romans were debtors to the Greeks. In fact the medical practitioners of ancient Rome were either Greeks or persons who had been educated in the schools of Greece. That *medici ocularii* were not wanting among the Romans, we have sufficient proofs in the inscriptions on seals, &c., which are to be met with in collections of antiques. Ophthalmiatry continued to be successfully cultivated and practised by the younger Greeks, and much on the diseases of the eye is contained in the works of the Arabian writers, derived no doubt from the Greek manuscripts which fell into their hands.

Until the commencement of the last century, little more was known of diseases of the eye than what is found in the Greek and Arabian writers; but as the anatomy of the eye began to be more carefully studied, so its diseases became better understood. Circumstances were, moreover, in a favorable train: Lord Bacon's philosophy had taught men the necessity of making experiments and observations;—the true seat of cataract which had been demonstrated by Rolfink, Borel, and others, was now confirmed by Brisseau. Moreover, Kepler had, by his discovery of the real use of the crystalline lens, proved the retina to be the true seat of vision, and had explained the action of glasses (invented three or four hundred years before) in improving sight.

It was in 1728 that Cheselden succeeded in first making an artificial pupil—an operation, the idea of which appears to have been previously suggested by Woolhouse, surgeon to James II. About the middle of the same century, Daviel, a French surgeon, practised extraction through an incision of the cornea, as a regular method of removing cataract; while a few years later, Percival Pott adopted laceration of the capsule and the breaking up of the lens as a distinct mode of operating independent of couching. It thus appears that the first grand improvements in eye-medicine were all made by English and French surgeons. Before the establishment of the Ophthalmic School of Vienna, eye-medicine was in so low a state in Germany, that those who could afford it went to France to be operated on for cataract. A complete revolution in this matter, however, has taken place since, and the Germans have far outstripped the French, and were fast outstripping the English surgeons, until the breaking out of the Egyptian ophthalmia in the army forcibly recalled the attention of the latter to the subject of eye diseases. More recently the French have begun to bestir themselves also. All that is now wanting, therefore, to impart to eye-medicine the highest possible state of perfection is to bring the energies of the English and French surgeons fully to bear on the subject, by rendering it a more important item in the curriculum of study than has yet been done in France and England; but which has already obtained for years in Germany.

As the establishment of the Ophthalmic School of Vienna forms an important era in the history of eye-medicine, and as it has had a most beneficial influence on its advancement, we think it will not be out of place here to give a short historical sketch of it, derived from Dr. Hardwiger's thesis.

In 1773 the Empress Maria Theresa established in the University of Vienna a special chair for eye-medicine. Barth, who was born in Malta in 1745, was the first professor. He was afterwards appointed to the professorship of anatomy in addition. On the establishment of the great General Infirmary in 1784 by the Emperor Joseph II., two large wards were set apart for eye-patients; and a great number of persons affected with cataract were collected from all parts by public advertisement. These Barth operated on and attended carefully until their recovery; and this was continued yearly until his resignation in 1791. In 1784 Barth selected, at the wish of the Emperor, two persons to be specially instructed in ophthalmic medicine and surgery—his choice fell on his own prosector Ehrenritter, and on the prosector at the Josephine Academy, the afterwards so-celebrated John Adam Schmidt. Barth was succeeded in his professorships of anatomy and ophthalmology by the distinguished Prochaska. Beer, born in Vienna in 1765, was, on account of his talent for drawing, taken by Barth to assist him in his microscopical researches and afterwards to be his private assistant. As Barth retired from practice Beer got into it; and in 1797 began to give private instructions in eye-medicine. After having taught in this manner for a period of fourteen years, a plan of public clinical instruction in eye-medicine was established by order of the Emperor in 1812. On the 28th of April of the following year, Beer commenced his clinical duties as extraordinary professor. In 1818 he was made ordinary professor, and the subject of eye-medicine was elevated to the rank of an ordinary branch of medical study. F. Jaeger and Rosas were Beer's pupils in 1812-13. Rosas was Beer's assistant from 1816 to 1819. In the year 1819 Beer was seized with apoplexy, and died in April, 1821. Rosas, who had been two years professor of ophthalmology in Padua, succeeded Beer. The conferring of this appointment upon Rosas in preference to Jaeger was considered owing to the operation of private interest, and not by any means to superiority of qualification in Rosas; for Jaeger was with justice pointed to by the profession as the proper successor of Beer. Jaeger, who, by the by, is Beer's son-in-law, is now professor in the Josephine Academy, the situation which John Adam Schmidt held.

Beer is well known by the accurate descriptions and histories of the diseases of the eye he has given in his works, and by the great reputation he acquired by his clinical prelections, which attracted students from all parts. His contemporary, John Adam Schmidt, though less generally known than Beer, was superior to him perhaps in originality and genius —a judgment which may be justly extended to their respective successors. To John Adam Schmidt eye-medicine is indebted for some most valuable contributions. He it was who gave the first correct account of *iriditis*, for, strange as it may appear, surgeons were unacquainted with the real nature of that disease until the publication in 1801 of his work on "Iritis and Secondary Cataract occurring after Operations for Cataract."*—Indeed, we may say that we owe most of our knowledge of the internal inflammations of the eye to the labours of German surgeons, though it must be confessed that they have occasionally refined too much in their distinctions. Mr. Ware, in his Treatise on the "Ophthalmy," never once hints at inflammation of the internal parts of the eye. He does not even seem to be aware of the fact that they are subject to inflammation—"a remarkable illustration," says Mr. Lawrence, "of the difference between seeing and observing disease, or rather a proof that the most obvious things will not be seen, unless persons know what to look for."

For the ophthalmic clinic in the General Infirmary at Vienna, there are two wards, each containing ten beds. The cases best adapted for clinical instruction are chosen by the assistant from among the daily general admissions. There is a museum and library attached to the ophthalmological institution. The library contains about 2000 volumes on medicine and surgery in general, but chiefly on eye-medicine.

Great pains are taken in the instruction of the students. Rosas gives two courses of lectures in the year: the one from the beginning of October to the end of February, and the other from the 1st of March to the end of July. At the termination of each course the students are examined for the space of two or three weeks. At the Clinic the students are instructed how to examine the patients; they have cases intrusted to them, reports of which they are required to give, and afterwards to draw up from them a complete history of the case, which is deposited in the archives of the Clinic. The visit, clinical instruction, and lecture, occupy three hours every day except Saturdays and Sundays. The hours are from 10 a.m. to 1 p.m.

Jaeger is Professor of Ophthalmology in the Josephine Academy—an institution for the instruction of those intended for the military service. Jaeger's public clinique is, therefore, in the military hospital. There are

* Ueber Iritis und Nachstaar nach Staar-operationen.

set apart for the purpose ten beds for males and ten for females, and an operating theatre. Jaeger has also a polyclinic at his own house, to which he readily and kindly admits visitors.

The private courses of the operative surgery of the eye, given by Jaeger and Rosas, are much attended by strangers, who go to Vienna for the purpose of visiting the ophthalmic school. Jaeger's courses are the more esteemed.

In speaking of eye-medicine in Vienna, the name of Burkard Eble, an army surgeon, must not be passed over in silence. His work on the Structure and Diseases of the Conjunctiva, published in 1828, and others which have appeared since, together with that at the head of this article, keep up the reputation of the school whence they emanated.*

Since the establishment of the Vienna School of Ophthalmic Medicine, the eye-clinic has become an essential part of the medical curriculum of every German University; and, indeed, the very language of Germany is so interwoven with the literature of eye-medicine, that he who would claim to be an authority in the latter must be well acquainted with the former.

The most celebrated names in connexion with German ophthalmic medicine and surgery, in addition to those just mentioned, are Richter, Himly, and Langenbeck of Göttingen; Walther of Munich; Graefe and Juengken of Berlin; Ammon of Dresden; Fischer of Prague; Fabini of Pesth; Benedict of Breslau; Beck of Freibourg; Chelius of Heidelberg; Ritterich of Leipzig—a long list, which, however, it would not be difficult to enlarge by other names of just celebrity.

In Britain the diseases of the eye have always attracted a considerable share of attention. Indeed, English surgeons have contributed as much to the real advancement of eye-medicine as those of any other nation. The establishment of the London Ophthalmic Infirmary has been an example, well followed up in many other parts of the kingdom. We may instance in particular the Eye Infirmary of Glasgow as being the source whence has emanated the standard work of Mackenzie, although this is not its sole recommendation; the systematic and efficient mode in which it is conducted, beneficial alike to the patients and students, calls for all applause.

In Italy, considerable attention has been bestowed on the subject of diseases of the eye, and in the schools there are ophthalmic clinics similar to those in Germany. Italian ophthalmological literature boasts of the works of Scarpa, Quadri, and others.

The name of Maunoir advantageously represents ophthalmology in Switzerland. Until very recently the School of Medicine of Paris, so famous for medical science in general, offered no adequate opportunity for the study of ophthalmic medicine and surgery, and French medical literature, so rich in other respects, boasted of no work of any merit on diseases of the eye. *On a changé tout cela,* however; and now, what from the labours of French surgeons themselves, and those of Germans and Italians settled among them, there are cliniques for the diseases of the eye, lectures on the diseases of the eye, and new books numberless on the diseases of the eye. The most recent of these works we intend to examine in this article.

* We regret to state that Dr. Eble is very recently dead.

Here we may allude to a contribution recently made to ophthalmoscopy —we mean the mode of ascertaining from the images, of a candle for instance, reflected by the eye, the state of the lens as regards its transparency or non-transparency, when the ordinary mode of examination may prove insufficient. Though this catoptrical mode of exploring the eye has come into notice only since M. Sanson published his ingenious observations on the subject, and though we believe these observations to be quite original on the part of M. Sanson, it is but justice to Professor Purkinje, of Breslau, to mention that he had, in 1823, made similar ones, and not only made a similar application of them, but accurately described them and illustrated them by figures in a small work entitled "Commentatio de examine physiologico organi visûs et systematis cutaneî quam die xxii Decembris, MDCCCXXIII, &c., publice defendit J. E. Purkinje, assumto socio G. Kraus. Vratislav. c. tabul. lith." 8vo, pp. 58.

Before concluding this historical sketch it will not be out of place to notice the first introduction of, and make a few remarks on, certain remedies of acknowledged efficacy in ophthalmic medicine.

1. Mercury. The use of mercury in diseases of the eye appears to have been long known, but, it must be confessed, only in a vague and general manner; and although it appears that in 1799, Beer (having been led to employ mercury in inflammation of the eye and its appendages on the recommendation of Warner), was acquainted in some degree with the power of mercury in obviating the effects of iritis, it is to be remembered that as the nature of iriditis had not then been properly defined, the indications for the use of mercury must have been very vague. We cannot, therefore, but consider ourselves indebted to Dr. Farre and Mr. Travers for having fixed particular attention on calomel and opium as a remedy in iritis.

2. Belladonna, Hyoscyamus, &c. The effect of certain vegetable substances in dilating the pupil appears from Pliny to have been known to the ancients, and advantage taken of it in operating on the eye. Darwin (*Zoonomia*, vol. iii., pp. 132—*London*, 1801,) suggests that the power of belladonna in dilating the pupil might be of advantage in some ophthalmiæ. The late Professor Himly, of Göttingen, however, was the principal means of drawing attention to the various useful applications of the artificial dilatation of the pupil in the diagnosis and treatment of diseases of the eye.

3. Disulphate of quinine. In 1763, Dr. Fothergill and Dr. Fordyce recommended a decoction of powdered bark as a remedy for scrofulous conjunctivitis. In the year 1828 Dr. Mackenzie was led by accident to try quinine in a very obstinate case of scrofulous ophthalmia. The patient, a child, was cured in a few days. Dr. M. afterwards derived advantage from the remedy in scrofulous corneitis and ophthalmia tarsi. Mr. Wallace of Dublin published in the same year a favorable account of the effects of cinchona in cases of iritis occurring after typhus fever; and Mr. Middlemore, of Birmingham, has communicated to the public the results of his employment of quinine in scrofulous iritis. "The treatment of the scrofulous ophthalmiæ with sulphate of quinine," says Mackenzie, "is an improvement in ophthalmic medicine, perhaps scarcely less important than the treatment of iritis with mercury." (p. 363.)

The remarks we have to make on the works before us we shall arrange under the three heads of Anatomy, Physiology, and Diseases of the Eye:

I. Anatomy of the Eye. In the general notice of Dr. Mackenzie's work in our last Number, we alluded to the Anatomical Introduction by Mr. Wharton Jones. M. Carron du Villards devotes seventy pages to a Physiological Anatomy of the Eye and its appendages. Although M. C. du V. pretends to be very elaborate and precise in the anatomy of the eye, and promises further great things on the subject, it must be confessed that the lengthy sketch before us is very indifferent. It deserves no particular notice—certainly no commendation. M. Rognetta does not give any set anatomical description of the eye, but there are interspersed throughout his book a number of anatomical and physiological observations. M. Jeanselme, the editor of M. Velpeau's lectures, judiciously gives, by way of introduction, a long extract from M. V.'s Traité d'Anatomie Chirurgicale on the distribution of the blood-vessels of the eye. Dr. Von Ammon, in his Essay on Iritis, devotes the first of six chapters to the anatomy and physiology of the iris. We can here notice only one or two of the more important points in the anatomy of the eye.

Mr. Wharton Jones distinguishes in the cornea three principal layers —the proper substance of the cornea in the middle, the conjunctiva corneæ in front, and the membrane of the anterior chamber of the aqueous humour behind. He denies that the proper substance of the cornea is composed of laminæ, but describes it as consisting merely of interweaving bundles of fibres. The appearance of a lamellar exfoliation, which the cornea sometimes presents in disease, is by some considered a proof of its lamellar structure. M. Rognetta, however, well remarks (p. 212), that the same argument has been advanced in regard to the structure of bone, and that the observations of Scarpa have proved that the structure of bone is not lamellar any more than that of the cornea.

"The conjunctival layer of the cornea, not admitting of being separated in a distinct form like the sclerotic conjunctiva, may be viewed as composing, with the proper substance of the cornea, a fibro-mucous membrane. What of the conjunctival layer of the cornea can be separated by the action of boiling water consists of a fine epithelium, together with a substance which may be compared to the corpus mucosum of the tegumentary system.........What is called the membrane of the anterior chamber of the aqueous humour or membrane of Descemet, lines the whole posterior surface of the cornea.........Its free surface is invested with a very delicate epithelium, composed of microscopical nucleated corpuscles.

"The three structures of the cornea just described have a close vascular connexion with each other. Individually, the conjunctival layer has a direct vascular connexion with the conjunctiva, the proper substance with the sclerotica, and the membrane of Descemet with the iris. Around the margin of the cornea, the vessels of all these parts communicate." (*Mackenzie*, p. 21.)

Henle has found epithelic corpuscles on the corresponding surfaces of the sclerotica and choroid, a confirmation, he thinks, of the opinion that a serous membrane (arachnoidea oculi) invests those surfaces. This opinion Henle attributes to Arnold, but it is one of long standing. In this country Wardrop revived it, and it is to be found expressed in the first edition of the Dublin Dissector, published some ten or twelve years ago, that is, four years before Arnold's work was published.

Viewed externally, the transverse diameter of the cornea is a little longer than the vertical; hence the circumference of the cornea is oval, and this in the strict sense of the word, for towards the temple it is smaller than on the side next the nose.

"Not exactly in its centre, but a little towards the nasal and upper side, the iris is perforated by the pupil..... ...The optic nerve enters the posterior part of the eyeball about one fifth of an inch to the nasal side of its axis." (*Ib.*)

The above are data calculated to assist in solving a question, suggested by M. Rognetta as one which might possibly occur in forensic medicine, viz., "an eye detached from the body being given, to determine to what side it belonged."

"Place the eye on a table," says M. Rognetta (p. 197), "with the cornea towards the observer. Then determine, by the exact measurement of the diameters, the upper side of the corneal disc: this side always corresponds with the short diameter. .. ...... This side is to be placed uppermost, as in the natural state." M. R. here comes to a conclusion too hastily; this observation can only determine that one of two sides must be the upper; but to determine which, the position of the pupil must be examined, and then we may, with M. R., "regard the insertion of the optic nerve. This nerve is not inserted in the centre of the sclerotica, but rather below and towards the nose." "Now," continues M. R., "a little reflection will tell us that if the insertion of the nerve of the eye in question is found nearer the right side of the observer than the left, the organ belongs to the left orbit, and *vice versa.*" (p. 197.) No! M. Rognetta. The organ belongs to the *right* orbit, and *vice versa.*

In our Number for October last, there will be found, among our selections (p. 580), a paper on the Physiology of the Iris, by Dr. Bolton, of Baltimore, extracted from the American Medical Intelligencer for 1838.

Dr. Bolton concludes that the idea of a dilator muscle of the pupil in the iris is incompatible with some of its most important phenomena. "Now," says he, "if we admit the radiating fibres to be elastic, we have an easy and satisfactory explanation of all the phenomena........... When the sphincter is passive, the pupil is given up entirely to the power of the elastic fibres of the iris which dilate it." In proof of an inherent elasticity of the iris tending to keep the pupil dilated, Dr. B. adduces the following experiment: Stretch the iris towards the pupil, and it will be found to resist, and to return to its former position immediately on being relieved from this state of tension. This is all true; but Dr. B. should not have stopped here, but should have ascertained whether, when the pupil is dilated, by inserting a closed forceps into it, and then allowing the legs of the forceps to separate, the pupil has not, in consequence also of the elasticity of the iris, a tendency to return to its former state. This is an experiment which was described by Mr. Wharton Jones in the Edinburgh Medical and Surgical Journal for January, 1834.

Dr. B. objects that the dilated state of the pupil can scarcely be an active state, which it must be if a dilator muscle is admitted, considering that in amaurosis the muscle must remain in the state of contraction from twenty, thirty, or forty years. This, it must be confessed, is a difficulty attending the admission that the great dilatation of the pupil is an active state. But a similar difficulty would exist in regard to the view that the only active state of the iris is that in which the pupil is more or less contracted, supposing it was well founded, which it is not.

As far as elasticity is concerned, Dr. B.'s view of the mode of dilatation of the pupil is the same as that held by those who consider that the contraction of the pupil is owing to an erectile structure of the iris. Thus, M. Rognetta, in speaking of the action of belladonna in the dilatation of the pupil, says: "If it is true that the whole arterial system falls into a sort of sunken state from the action of belladonna, it is evident that the very vascular organs are those which will be most subjected to the effects of this substance. The iris in fact experiences a very strongly marked relaxation. The elasticity of the tissue of the iris being no longer counteracted by the arterial erethism, that membrane collapses, retracts, and the pupil thus becomes dilated. It is commonly said," continues M. Rognetta, "that belladonna paralyses the iris. That is not correct; true paralysis of this partition is not accompanied by dilatation of the pupil; its substance is then, on the contrary, relaxed and vacillating like any other paralysed tissue." (p. xv.) This observation is correct; but it is incompatible with his opinion that the contraction of the pupil depends on an erectile structure, and its dilatation on the mechanical retraction of the elastic tissue set free by the subsidence of the erethism.

The views of both Dr. Bolton and M. Rognetta are one-sided. We believe the following extract from the English author already quoted, will be found a correct account of the state of the case as far as is yet known. According to it, it is to be remarked, that belladonna must produce dilatation of the pupil by directly or indirectly exciting to contraction the radiating fibres of the iris.

"Besides blood-vessels and nerves, in which the iris is very rich, another tissue enters into its composition, which, in order to explain the motions of the iris, is admitted to be muscular, though it does not exactly resemble even the unstriped organic muscles of other parts, much less the characteristically striped fibres of common muscles. The iris, it may be remarked, being suspended in a watery medium, less power will be required to move it than if it were suspended in air, in consequence of the resistance of its own weight being thus in a great measure removed. The notion of the structure of the iris being erectile is disproved by all the phenomena attending its motions. It is certain that when there is dilatation of the pupil, the iris is as much in an active state as it is when there is contraction of the pupil. In the former case, the larger ring of the iris is contracted in the direction of its radius, and in the latter case the smaller ring is circularly contracted. It is also certain that the state of relaxation of the iris is that in which the pupil is neither much contracted nor much dilated, a state in which the pupil always is sometime after death, and to which, in consequence of an elasticity which the tissue of the iris possesses, it has a constant tendency to return after the contracting or dilating force has ceased to act." (*Mackenzie*, p. xxv.)

Just twenty years ago, Dr. Jacob, of Dublin, made known the discovery of a very delicate membrane situate between the retina and choroid. Though Dr. Jacob both delineated and described the membrane well, it was not generally admitted; it and two other structures, viz., the membrane of the pigment and the tunica Ruyschiana being frequently confounded. Since the publication of Mr. Wharton Jones's paper, however, on the Membrane of the Pigment, in the Edinburgh Medical and Surgical Journal for 1833, correct views of these three parts of the eye have been gradually making way. "Anteriorly," says this writer, "the choroid may be separated into two layers; but towards the back part of the eye such a separation is impossible. The division of the choroid into two layers was first mentioned by Ruysch, whose son, Henry, proposed the name of *tunica Ruyschiana* for the inner, leaving to the outer the name of *choroid*." Such is the tunica Ruyschiana. The membrane of the pigment, known to John Hunter, Carlo Mondini, and Kieser, was more completely investigated and extremely well described by Francesco Mondini in 1818. Notwithstanding this, Döllinger appears to have confounded it with the membrane of Ruysch, and others confounded it with the membrane of Jacob. Mr. Wharton Jones, by discovering its remarkable microscopical appearance, has secured for it a distinctive character. The microscopical appearance of the membrane of Jacob having also been delineated by him, and more recently by Professor Valentin, of Berne, there is now no excuse for denying the membrane of Jacob, but want of dexterity to demonstrate it.

The intimate structure of the retina is thus described, according to the most recent observations:

"The primitive fibres or tubules of the optic nerve are entirely microscopical. They are similar to those constituting the substance of the brain, with which they are, on the one hand continuous, and, on the other, with similar fibres in the retina. In addition to its blood-vessels, and the delicate cellular tissue supporting them, the retina presents three different elements in its structure, forming as many layers. The outermost is a mucus-like substance, consisting of granules or globules arranged in the manner of pavement. The next or middle layer is formed of the radiating expansion of the primitive fibres or tubules, continued from those of the optic nerve. The third or innermost layer, which is found over the whole inner surface, the entrance of the optic nerve not excepted, consists of upright cylindrical papillary bodies, which project through the vascular layer, and into which the fibres of the middle layer, suddenly bending inwards, are supposed to pass. The cylindrical papillary bodies are difficult of demonstration, as they are very brittle, readily separate from the retina, and become changed. The fibrous layer of the retina is most easily shown in the rabbit." (*Mackenzie*, p. xxviii.)

A remark or two more in regard to the surfaces of the aqueous chambers, and we have done with the anatomy of the eye.

In his Chapter on the Anatomy of the iris, Dr. von Ammon says that he has in no case been able to observe the serous membrane on the posterior surface of the iris. We are surprised at this, for a membrane lining the posterior chamber of the aqueous humour is more demonstrable than one lining the anterior chamber throughout. The membrane on the posterior surface of the iris was, many years ago, described and delineated by Dr. Jacob, of Dublin, in the Medico-Chirurgical Transactions.

"The free surface of the membrane of Descemet is invested by a delicate epithelium. Henle has found an epithelium neither on the surfaces of the iris nor on the anterior wall of the capsule of the lens, a new proof, says he, against those who think the whole anterior and posterior chambers are lined by a serous sac. Valentin, however, describes an epithelium, both on the anterior and posterior surfaces of the iris; and I can add that I have found epithelic corpuscles on the anterior surface of the anterior wall of the capsule of the lens in a lamb's eye." (*Mackenzie*, p. xxxi.)

Dr. von Ammon broaches a new doctrine in regard to the secretion and absorption of the aqueous humour, viz. that the serous membrane investing the anterior surface of the iris is villous, and secretes the aqueous humours, whilst the absorption of it is the work of that part of the serous membrane of the anterior chamber lining the cornea.

M. Rognetta appears to have a favorite notion that the tears are derived principally from the aqueous humour oozing through the cornea.

He harps upon this in many different parts of his book, and is very sore at certain remarks which Velpeau appears to have made in regard to it. One argument employed by M. Rognetta is, that the lachrymal gland is too small to furnish all the tears. Taking the upper and lower masses of the lachrymal gland with their ten or twelve ducts, it seems to us, that in proportion to its secretion, the lachrymal gland is as large as any other.

II. Physiology of the Eye. The following remarks on this subject have been suggested chiefly by a perusal of the section on Vision, in Part V. of Müller's Elements of Physiology, Dr. Alison's paper on Single and Correct Vision, by means of double and inverted images on the Retinæ, in the Transactions of the Royal Society of Edinburgh, Mr. Wheatstone's "Contributions to the Physiology of Vision," in the Philosophical Transactions, Part II. for 1838, and several papers in Müller's Archives, by Professors Volkmann and Mile.

Passing over the first two Chapters of Müller's Section on Vision, which treat the one of " the Physical Conditions of Images in General," and the other, "of the Eye as an Optical Instrument," we come at once to Chaper III. which is on "the Actions of the Retinæ, Optic Nerve, and Brain in Vision," a part of the subject of great importance, not only in a philosophical but also in a practical point of view. By adopting this arrangement, we shall have space and opportunity to bestow that attention on the memoirs of Dr. Alison, and of Mr. Wheatstone, which their value and importance demand.

"All the phenomena," says Müller, (Baly, 1162,) "investigated in the preceding chapter, are explicable by reference to the structure of the eye as an optical instrument,—that is, by the form and arrangement of the transparent media in front of the retina. There are a great number of other phenomena, however, of which the structure of these parts affords no explanation, but which are the results of vital properties of the retina, and of the cooperation of the sensorium in the act of vision. To these belong not merely the act of sensation itself, and the perception of the changes produced in the retina, as light and colours, but also the conversion of the mere images depicted on the retina, into ideas of an extended field of vision,—of proximity, and distance,—of the solidity (in the geometrical sense) and size of objects. To this class of phenomena belong also the effects of the reciprocal action of different parts of the sensitive apparatus on each other, and many phenomena in the retina, either not excited by light at all, or not by its immediate action."

In studying the physiology of vision, it is of great consequence to have a clear idea at the outset, of what are strictly physical, and what are strictly physiological questions; for the subject of vision has been much embroiled by the attempt which has been often made to explain purely physiological points on physical principles.

One of the most difficult problems in physiology is that relative to the respective influence of the retina and sensorium in vision.—"Where," asks Müller, "is the state of the retina perceived;—in the retina itself, or in the brain?"

"In the present state of our knowledge," answers Müller, "it is utterly impossible to solve this problem.

"However the above question may be decided, it is at all events certain, that even after the retina and external portion of the optic nerve are lost (as after extirpation of the eye), the internal or central portion of the organ of vision is capable of exciting not merely sensations of light, but also the same ideas of a field of vision, in which images are perceived as when the retina is present. . . . . These facts (lights, figures, &c., floating in front of the orbit from which the eye has been removed) would seem to prove that the affections of the fibres of the optic nerve are communicated to the brain before they give rise to the sensation of a field of vision; and, in that case, we must admit that each minute division of the retina is represented in the sensorium by a corresponding nervous fibre, though this cannot be demonstrated anatomically.

"The mode of action of the peripheral and central parts of the apparatus of vision upon each other is, therefore, at present, involved in much obscurity; and we must rest satisfied with a knowledge of the fact, that the order of the images in the field of vision depends upon the relative position of the affected parts of the retina." (*Baly's Müller*, 1165.)

We shall confine our further remarks on Müller's chapter on the "action of the retina, optic nerve, and sensorium in vision," to two points, viz. "inverted images, and erect vision," and "single vision with the two eyes," which have always been a sort of stumbling-block in the physiology of vision. The memoir of Dr. Alison, we think, is calculated to throw some light on the former point, and that of Mr. Wheatstone on the latter.

Müller's observations on the subject of inverted images and erect vision, are—since the image and the affected parts of the retina mean the the same thing, the question physiologically expressed is this:

"Are the minute divisions of the retina affected in vision perceived by the sensorium in their natural relation to the object? The view which I take of the question," adds Müller, "and which I proposed in my work on the physiology of vision, is, that even if we do see objects reversed, the only proof we can possibly have of it is that afforded by the study of the laws of optics; and that, if every thing is seen reversed, the relative position of the objects of course remains unchanged." (*Baly*, 1171.)

"The difficulty," says Alison, "which is presented by the inversion of the images on the retina is, I think, most correctly expressed thus: the sensations both of sight and touch obviously differ from one another in position, and by doing so, both convey to us intimations of the situation of external objects. But the judgments which we form of the relative position of objects, or of the parts of an object, from the relative position of the impressions which they make on the sensitive surface of the *retina*, are just the reverse of those which we form of the relative position of objects or their parts, from impressions made on the sensitive surface of the *skin*. Thus, if two impressions are made on the upper and lower portions of the eyeball, and felt through the fifth nerve, the inference immediately drawn is, that the upper impression is from a higher object, and the lower from a lower; but if two impressions are made in the upper and lower part of the retina, and felt through the optic nerve, the inference is, that the impression on the upper part is from the lower object, and that on the lower part, from the higher. Why this difference should exist, is the point in question."

These observations of Alison, clearly prove the unsatisfactory nature of the view above expressed, which, by the way, is not that of Müller only. As to the explanation of erect vision with inverted images on the retina, by reference to what is called the "Law of visible direction," we would remark, that the perception of the impression on the retina is one thing, and the direction in which the rays of light are transmitted to the retina another, necessarily associated, indeed, but not essentially related.

"The explanation which seems to me satisfactory," says Alison, "of the erect vision by inverted images, was first suggested to me by Mr. Dick, veterinary surgeon, and turns on the alleged fact, that the course of the optic nerves and tractus optici is such, that impressions on the upper part of the retina are, in fact, impressions on the lower part of the optic lobes, *i. e.*, of the sensorium, and impressions on the outer part of the former are on the inner part of the latter, and *vice versa.*"

This view of the matter, which also appears to us satisfactory, is supported by the following considerations:—The experiments of Flourens repeated before and reported on by Cuvier, are generally regarded as affording satisfactory evidence that the sensations of the eye reside in the corpora quadrigemina.

"Our business is," says Alison, "to learn in what manner those fibres of the tractus optici, which can be distinctly traced into the corpora quadrigemina, are there implanted; and when we trace the course of these fibres in the brains of the mammalia, (hardened by alcohol,) whether they descend on the corpora quadrigemina from the thalami, or pass more directly backwards below the corpora geniculata, it seems to me quite obvious, that they first turn inwards, and then enter the corpora quadrigemina from above downwards, and are so expanded over the superior of these bodies (the nates), that the outer portions of the tractus pass over to the inner part of the nates, and the upper portions of the tractus pass down to the lower part of the nates. . . . . . . . . Now, there is no such contortion or involution of the nervous filaments of the fifth, or of any other nerve of the symmetrical system, where it is implanted in the cerebro-spinal axis, and so constituted a nerve of touch; and from this I think it clearly follows, that although the impressions made on the *retina*, by the different parts of an object, are situated in regard to one another in the *inverse* order of those made on the surface of the body, yet the impressions made, *through the retina* and optic nerves, on the *cerebro-spinal axis*, are *in the same order* as those made through the nerves of touch, on that central portion of the nervous system, on which the sensibility of all nerves depends; and therefore, that the notions which we form of the relative position of the parts of objects, by the senses of sight and of touch, will naturally correspond."

In opposition to the view now stated, the following observations from Müller might be advanced:

"The inversion of objects," says he, "being a thing of which we can never become conscious in ourselves, it is not possible that nature has made in the brain, or elsewhere, any provision for the correction of the error, which would never have been known but for the institution of optical enquiries. The decussating course of the optic nerves cannot be adduced as an explanation of erect vision, since the decussation is only partial." (*Baly*, 1179.)

This is, no doubt, true; but still we reply, the decussation along with the twisting of the optic nerves may be adduced as an explanation. "If it were possible," continues Müller, "to produce an image of an object upon the retina without the aid of light; for instance, by immediate contact, the image would in that case not be inverted." The image on the retina would not be inverted; but, on that very account, the mind would acquire the idea that the impressing object was inverted.

"If it were possible," Müller farther continues, "to see the same object simultaneously by means of luminous rays from without, and by immediate impression upon the retina, the images produced in these two ways would appear to lie on opposite sides. This can, in fact, be shown experimentally. If we press upon the retina with the finger through the sclerotica, a spectrum produced by the immediate impression of the finger will be perceived, while at the same time the finger may be seen through the medium of the external light. The two images will appear at opposite sides. If, while the eyes are closed in the dark, we press upon what appears to us to be the upper part of one eye with the finger, which is, therefore, seen above, the spectrum produced by the pressure becomes visible below; if the pressure be made upon the lower part of the retina, the spectrum appears above; if the right side of the retina be the seat of the pressure, the spectrum appears on the left side, and *vice versâ*." (*Baly*, 1172.)

In these experiments it is different parts of the retina which are excited. The image of the finger produced through the medium of the external light falls, when the finger is held above, on the lower part of the retina; and in consequence of the constitution of the optic nervous apparatus, the finger is seen where it is. In the case of images by pressure, they are seen in the place where they would be if the impressing agent were sent in the ordinary manner, the optic nervous apparatus being arranged and constituted in conformity with the circumstance, that it receives impressions, not by the direct contact of the object, but, through the intermedium of an agent which is conveyed by an apparatus corresponding to its peculiarities, in a manner the inverse of what takes place in the perception of objects by immediate contact. And it is to meet this peculiarity that the optic nerves, as has been already said, are so disposed by semi-twisting on their axis at their origin, and semi-decussation, that an affection of the upper part of the retina is communicated to the lower part of the cerebral portion of the optic apparatus, and so forth. We think it of importance to remark here, that, according to Müller's exposition of the structure of the compound eyes and mode of vision of insects and crustacea, it is obvious that the image painted on the retina of those animals will not be *inverted* and *reverted*, but like the image in a mirror, *erect*.

We now come to single vision with two eyes; or, in other words, a single visual perception from two retinal images.

Some physiologists have sought to explain this by supposing, that we really do not employ both eyes simultaneously in vision, but always see with one only at a time. Müller rejects this opinion, and maintains what indeed is generally admitted, viz., that single vision results only when certain parts of the two retinæ are affected simultaneously; if different parts of the retinæ receive the image of the object, it is seen double. The parts, the simultaneous affection of which is found to yield a single perception, are called identical or corresponding, and are the various points of the right and left sides of the two retinæ equally situated in reference to the axis of the eyes.

"The cause of the impressions on identical points of the two retinæ," says Müller, "giving rise to but one sensation, and the perception of a single image, must lie in the organization of the deeper or cerebral portion of the visual apparatus; it must, at all events, depend on some structural provision, for it is the property of the corresponding nerves of the two sides of the body in no other case to refer their sensations as one to one spot. It is exceedingly improbable that the identical action of the corresponding parts of the two retinæ is the result of a certain habituation, or of the influence of the mind." (*Baly,* 1196.)

After enumerating the many attempts which have been made to explain this remarkable relation between the eyes, Müller (Baly, 1200) repeats his conclusion, that the cause of the single sensation, excited by impressions on identical points of the two retinæ, must be some organic or structural provision. "There are," he says, "many theories, involving the supposition of such a structure, which would account for the phenomena; but not one of these theories can be proved to be the correct one, and, with regard to several of them, it can be shown that they are certainly erroneous."

In reference to the opinion that the perception of the number and position of visible objects is acquired only by association or custom, Dr. Alison remarks, that this view of the subject had been previously fully considered and completely set aside by Dr. Reid, at least in reference to single vision by two images on the retinæ, and that, not by any abstract reasoning, but by appeal to facts.

"From the time," says Dr. Reid, "we are capable of observing the phenomena of single and double vision, *custom* makes no change in them. I have amused myself," he adds, "with such observations for more than thirty years; and in every case, wherein I saw the object double at first, I see it so to this day, notwithstanding the constant experience of its being single. In other cases, where I know there are two objects, there appears only one, after thousands of experiments. Effects produced by habit must vary, according as the acts by which the habit is acquired are more or less frequent; but the phenomena of single and double vision are so invariable and uniform in all men, and so exactly regulated by mathematical rules, that I think we have good reason to conclude that they are not the effect of custom, but of fixed and immutable laws of nature."*

Dr. Alison concludes, that *images formed on corresponding points of the retinæ of the human eyes, and on those only, naturally affect our minds in the same manner as a single image formed on the retina of one eye*, is an established fact, not to be explained by experience or association; and, not being *necessarily* an ultimate fact, affords a fair subject of physiological enquiry.

Dr. Alison then proceeds to illustrate this position by anatomy, in a manner much the same as is done in the work of Müller. The latter also answers the objections to the doctrine of corresponding points of the retinæ in a similar way with the former author.

The following is the opinion recently given by Professor Volkmann, one of the best modern authorities on the physiology of vision in Germany:

"Mile (Archiv, 1838, p. 387,) incorrectly says that single vision is only the effect of habit. The theory, that identical places of the two retinæ must necessarily see single, different places on the contrary must see double, has been recently completely confirmed by the important observations of Hueck (Die Achsendrehung des Auges, Dorpat, 1838, 4to.) Hueck affirms that the image of a perpendicular line immediately separates into a double image, the lines crossing, when we, by proper pressure of the finger, roll somewhat one eye. This assertion I can confirm from my own experiments. The business of the

* Reid's Works.

oblique muscles is, on every motion of the head, to maintain the original position of the eyes." (*Volkmann, in Müller's Archiv.* No. iii. 1839, pp. 240-1.)

Against the doctrine of corresponding points of the retinæ Mr. Wheatstone has lately offered very powerful objections. He has shown, in a manner at once convincing and ingenious, that in viewing an object of three dimensions, i. e., having length, breadth, and thickness, the perspective projections upon the two retinæ differ according to the distance at which the object is placed before the eyes; if it be placed so distant, that to view it the optic axes must be parallel, the two projections are precisely similar; but if it be placed so near, that to regard it the optic axes must converge, a different perspective projection is presented to each eye; and these perspectives become more dissimilar as the convergence of the optic axes becomes greater. Notwithstanding this dissimilarity between the two pictures, which is in some cases very great, the object is still seen single, though it is to be remarked not exactly resembling either of the two pictures on the retinæ.

"It being thus established," says Mr. W. (p. 373), "that the mind perceives an object of three dimensions by means of the two dissimilar pictures projected by it on the two retinæ, the following question occurs: What would be the visual effect of simultaneously presenting to each eye, instead of the object itself, its projection on a plane surface as it appears to that eye?"

For this purpose Mr. Wheatstone invented an instrument which he calls a *stereoscope*. It consists of two plane mirrors, with their backs inclined to each other, at an angle of 90°, near the faces of which the two monocular pictures are so disposed, that their reflected images are seen by the two eyes, each looking into one of the mirrors, in the same place.

The observation may be sufficiently well made by viewing the subjoined figures,—the dissimilar perspectives of a truncated four-sided pyramid, in the following manner:

Fixing the right eye on the right-hand figure, and the left eye on the left-hand figure, hold between the eyes in front of the nose the board of an octavo book. The two figures will be seen to approximate, and then run into one, representing the skeleton of a truncated four-sided pyramid in bold relief.

"Were the theory of corresponding points true," Mr. Wheatstone remarks (p. 392), "the appearance should be that of the superposition of the two drawings; to which, however, it bears not the slightest similitude."

The above experiment is decisive in the disproof of the doctrine as stated by Alison, that images formed on corresponding points, and on those only, naturally affect our minds in the same manner as a single image formed on the retina of one eye, but we cannot admit with Mr. Wheatstone that it disproves the doctrine of corresponding points altogether. And we find that in a paper recently read before the Royal Society, Mr. Wharton Jones endeavours to show that, though the correspondence of the two retinæ is not limited to *points*, there are compartments of the two retinæ having certain limits, of which any one point or papilla of the one corresponds with any one point of the other, so far that impressions on them are not perceived separately ; or, rather, that of impressions on them one only is perceived at one instant of time. In the same paper Mr. W. J. describes as a principal cause of the perception of relief or of intaglio, in consequence of two dissimilar perspectives of the object impressed on parts of the retinæ within each other's influence, an oscillation of great velocity of the mind between the two. He also conjectures that the central folds of the retinæ have something to do as a condition, central folds being found only in the eyes of man and the quadrumana, the axes of which alone can converge.

"There are some facts," says Mr. Wheatstone (p. 386), "intimately connected with the subject of the present article, which have already been frequently observed. I allude to the experiments first made by Du Tour, in which two different colours are presented to corresponding parts of the two retinæ. If a blue disc be presented to the right eye, and a yellow disc to the corresponding part of the left eye, instead of a green disc which would appear if these two colours had mingled before their arrival at a single eye, the mind will perceive the two colours distinctly, one or the other alternately predominating, either partially or wholly over the disc. In the same manner the mind perceives no trace of violet when red is presented to one eye and blue to the other, nor any vestige of orange when red and yellow are separately presented in a similar manner. These experiments may be conveniently repeated, by placing the coloured discs in the stereoscope,* but they have been most usually made by looking at a white object through differently coloured glasses, one applied to each eye."

Farther on (p. 391), Mr. Wheatstone says:

"Du Tour held that though we might occasionally see at the same time with both eyes, yet the mind cannot be affected simultaneously by two corresponding points of the two images. He was led to this opinion by the curious facts already alluded to in § 14," (the passage just quoted).

The phenomena just described as first observed by Du Tour, and discussed by Müller, under the head of "Alternate Predominance of the Sensations of the two Retinæ," appears to us calculated in some degree to reconcile Mr. Wheatstone's experiments with the doctrine of corresponding points, though we are aware that ingenious observer and experimentalist is of a different opinion. In the communication to the Royal Society already alluded to, Mr. Wharton Jones considers that Du Tour's experiments essentially prove that impressions on corresponding papillæ of the two retinæ cannot be perceived by the mind at the same instant of time, but sometimes the one, sometimes the other, or the

* The experiments under consideration may be readily repeated without the stereoscope, by viewing two different coloured wafers in the manner recommended for viewing the figures given above.—*Rev.*

stronger to the exclusion of the weaker, and that this is one of the causes of single vision with two eyes.

The observations of Professor Volkmann on the sensation which arises when different coloured rays of light fall on the same place of the retina of one and the same eye, though objected to by Professor Mile of Warsaw, we consider of much interest, and think they go far to illustrate what has just been related concerning the sensation arising from different impressions on the two retinæ.

Having allowed light of two different colours to fall on one place of the retina of one and the same eye, Volkmann found to his astonishment that a mixture of colours did not occur. The following are the results of his experiments:—

"When two different colours meet on the same place of the retina of one eye, it is found:

"1. That often only the one of the two colours appears without any transition into a mixed colour.

"2. Even when a certain mixture of the colours does take place, still a perfect middle colour, such as is obtained by the mixture of pigments, never appears, but we see one of the two colours with a tendency to the middle colour, and of a dirty tint.

"3. If we see only one of the two colours, even when it appears clean, it is still not so conditioned as it would be if no other colour was at the same time affecting the retina.

"4. If one colour only is seen, we perceive, *a*, the brighter of the two colours, especially when the brightness is combined with a glistening appearance; *b*, the colour of the object fixed; *c*, the colour to which the attention is directed."

III. DISEASES OF THE EYE. We propose first to notice, individually, but in a general manner, the different works before us on the Diseases of the Eye, and then to bring together such facts and opinions as appear worthy of consideration, and tending to give additional illustration to any particular point.

In noticing the different works, we shall take them as they stand chronologically arranged at the head of this article.

We are much pleased with the work of Dr. Andreæ, both as a literary composition and as giving an excellent elementary view of the subject of ophthalmic medicine and surgery.

To complete Dr. Ammon's large illustrated work on the Diseases of the Eye, a third Part is still wanting. It will contain the congenital malformations of the eyeball, the eyelids, lachrymal organs and orbits. To appear along with this third Part, the author promises a clinical exposition of the whole of ophthalmic pathology. The work, Dr. Ammon informs us, is intended to fill up a blank in ophthalmological literature, felt both by teacher and scholar. It aims at giving not only a systematic delineation of the external appearance of the diseases of the eye, but also figures illustrative of its "pathic histology." This has been in some degree the object of preceding authors, but it has been effected only to a very limited extent; even Wardrop's work is rather a series of pictures of the external appearances of the diseases than of the morbid anatomy of the eye. The book before us would not bear being criticised as a work of art, but the delineations are sufficiently good for the purpose. It may be observed that it is rare to find eyes well drawn. Well engraved as Demours's figures are, they are contemptible as representations of diseased eyes, and still more contemptible as drawings. More beautifully executed as Wardrop's figures are than any others, they are still by no means perfect. Even the specimens of eyes given in the first table of S. T. Soemmering's work on the Anatomy of the Eye are not accurately drawn. In portraits the expression of the eye is all that is required. Details in the delineation would rather injure the effect in general. In the portraits painted by Peter Denner, however, remarkable for the minuteness of detail and for the striking representation of texture, the eyes are delineated with a degree of accuracy and truth altogether unique.

While on the subject of artificial representations of the diseases of the eye, we would allude to the series of wax preparations belonging to the Ophthalmic School of Vienna, modelled from nature by Hofmayer, under the superintendency of Professor Rosas. They are very beautiful and expressive. Each cost seven guineas; so that for the whole collection of sixty, the Austrian government paid 420 guineas.

Though the particular history of the morbid preparations represented in Dr. Ammon's work is not given, but only a general description, the book will always be valuable as one of reference. The morbid changes which occur in the eyes of the lower animals, and what can be ascertained by experiments on them, are frequently called in to aid the explanation of points in the pathology of the human eye.

The indefatigable author of the above work, as will be seen from our list, has also published a Monography on Iritis, to which was awarded the prize offered by the Society of Practical Medicine of Paris.

Dr. Carron du Villards offers his book as a purely practical one to practitioners and students; and Dr. Rognetta tells us his is written in a style almost aphoristical, it having been his wish to economise space and time, without, however, being obscure or omitting anything essential.

Men of information, as Carron du Villards and Rognetta certainly are, their works appear to have been thrown together rather than maturely digested. Both have brought to the task a certain amount of theoretical knowledge and practical experience; but it must be confessed that the former, without being behind his rival in science, evinces more of the knowledge of detail, and, as far as can be judged from the mere perusal of a book, more of the practical tact indispensably necessary for the successful practice of the surgery of the eye. Better informed of the doings in other countries than their adopted countrymen generally are, both M. Carron du Villards and M. Rognetta, nevertheless, too often misunderstand what they are speaking of, and consequently are guilty of over-hasty criticisms, especially against the Germans. So much is this the case, that a German reviewer retorts on Rognetta, and, we think, justly, "Ars autem nostra non habet osorem nisi ignorantem." In regard to Carron du Villards, the same German reviewer remarks that he deserves blame for neglecting to acknowledge the source whence he has derived much that is contained in his book; "a detestable practice," he adds, "becoming more and more common, especially among the French."

The part of Professor Chelius's work before us is published both in the German and French languages. The French translation is, with the approbation of the author, by MM. Ruef and Deyber, of Strasbourg. The volume treats of the Organic Diseases of the Eye.

On account of their fixedness of character, organic diseases admit of being more easily and accurately described than the more protean dynamic diseases. Professor Chelius, in his well-known Manual of Surgery, does not enter into the diseases of the eye and ear, saying in his preface: "That I have not taken up the diseases of the eye and ear will not be brought against me as an objection, for the field of ophthalmic medicine and surgery has been so much extended as to require a particular work, and as I, like many other teachers, give separate courses on the diseases of the eye and ear." To those who are acquainted with the Professor's Manual of Surgery, it is enough to say that the volume on the Organic Diseases of the Eye before us is composed in the same style, that is, it is a judicious and comprehensive abstract of the present knowledge on the subject, and as such, well adapted to the purpose for which it is published, viz., for the use of students attending the author's lectures.

Dr. Eble's work on the so-called Contagious or Egyptian Ophthalmia, was written to compete for the prize of 1000 roubles, offered, in 1836, through the Society of German medical men at St. Petersburg, by a gentleman who had himself suffered from the disease. There was only one other work sent in, but the prize was given to neither. We have already had occasion to consider Dr. E.'s views regarding the Egyptian ophthalmia. We may mention that his opinion, that this disease is a mere modification of catarrhal ophthalmia, was objected to as a preconceived notion, and was what led principally to the refusal of the prize to it. The reporters admitted the excellence of the work in many respects. We believe it to be the most complete monography on the subject. There is a point which appears to us not always to be taken into account in the adjudication of prizes, and that is, whether the knowledge of the judges on the particular point, supposing their perfect impartiality, be such as to enable them to determine correctly.

Of Mr. Middlemore's Introductory Lecture we may remark that it contains, at pp. 10-11, an explanation of the method he has adopted to observe and describe the visible external symptoms of the diseases of the eye. The diseases of the eye form a good subject on which to exercise young men in the art of observing; and the method chalked out in the address before us is well worthy of being followed.

Passing over the work of Mackenzie, which we noticed in a general way in our last Number, we come to the manual which M. Jeanselme has composed from the lectures and published works of M. Velpeau. M. J. explains his double object to be "to make known the views regarding the diseases of the eye professed by M. Velpeau, and to initiate students and young practitioners into the practice adopted by that surgeon."

Of the general spirit of M. Velpeau's work, for we must view it as his work, we can speak in no other than terms of unqualified disapprobation. Not so much because he inculcates bad practice, but because, modifying the nomenclature in common use, and misrepresenting the current doctrines, he arrogates to himself the character of delivering new and more correct views of the pathology and therapeutics of the eye.

When he endeavours to vindicate France from the reproach often made to her, that she has contributed but little to the advancement of ophthalmology, we, to some extent, go along with him; but when he speaks as he does, in a somewhat contemptuous manner, of some of our most esteemed medical practitioners and authors as mere *oculists,* we cannot but regret the ignorance, wilful as it cannot but be, which should have allowed M. Velpeau to utter such an impertinency. Notwithstanding what we have said, however, we think the work is calculated to be useful to French students, and we must say M. Velpeau is fortunate in having so judicious an editor of his views as M. Jeanselme.

The volume of M. Vidal's Surgery before us is almost entirely occupied with the diseases of the eye. This circumstance, and the respectable manner in which the subject is discussed, together with the appearance of the works of Carron du Villards, Rognetta, and Velpeau, we hail as good indications of a rising interest among the French surgeons for the subject.

The methodical physical examination of the organ affected in any diseases, as far as that can be done, ought always to be a leading point in clinical instruction. No organ of the body, perhaps, offers greater facilities for this, or a greater variety of shades of difference to be noted, than the eye. Exercises in *ophthalmoscopia*, therefore, we hold would form a most valuable introduction to the physical investigation of diseases in general. Inasmuch, as regards the diseases of the eye itself, methodical examination is of the greatest consequence. Hence it is that ophthalmoscopia has, in some works, separate chapters devoted to it. Not long ago, we had occasion to notice an Italian work exclusively devoted to the subject. Attention to ophthalmoscopia is the most striking circumstance to be noticed in the ophthalmic clinics of Germany, and what has contributed so much to the general diffusion of a correct knowledge of the diseases of the eye. "It is of great moment," says Dr. Mackenzie, "to examine the diseased organ carefully and thoroughly from time to time; in some cases daily, or even oftener. Many children lose their sight in the puro-mucous ophthalmiæ, no examination of their eyes ever being made, till the corneæ are destroyed. The oculist never declines the examination of the eyes from any real or fancied difficulty." (p. 358.)

Dr. Andreæ gives a short but very sensible chapter on the "Examination of Diseased Eyes." M. Carron du Villards devotes thirty-two pages to "Ophthalmoscopia, or general rules and precautions to be observed in the examination of diseased eyes and their appendages;" and on the whole, the article contains a good and judicious summary of advice. M. Vidal has contented himself with extracting the principal points in M. Carron du Villards' observations. Mr. Morgan, in the work we formerly noticed, gives some useful hints to the young surgeon as to the necessity of handling gently, and with management, an inflamed eye when making the examination of it. "It may appear absurd," says Mr. M. (p. 24), "to suppose that any medical man in his senses would throw a stimulating injection into the eye, as a preparatory step to separating the lids, and obtaining in that way a view of the inflamed globe (in order to a diagnosis). Yet many are producing now and then the very same effect as that which would result from such a proceeding by different means. This effect is occasioned by the manner in which many a novice is in the habit of opening an inflamed eye. I speak of what I am not unfrequently witnessing in my practice; and as I know how few of you are acquainted with the proper mode of examining an inflamed eye, I shall take this opportunity of giving you a few directions on the subject." Mr. M. then proceeds to describe and illustrate by diagrams the right way and wrong way of exploring eyes, and concludes with the following just remark. "You may perhaps think that I have laid great stress upon a comparatively trifling subject; but experience will prove to you that minor points in your surgical practice are of more importance than you may now suppose." (p. 26.)

The eye in fact should be first examined without touching it. The eyelids may be then separated with the precautions only to be learned by experience and good example. A point often very necessary to ascertain, is the degree of firmness of the eyeball. This is of course done by touching and pressing with the finger or thumb, (*palpation ou toucher oculaire* of the French works before us.)

The catoptrical method of investigating the state of the crystalline body, as to its transparency or opacity, when there exist any doubts after the usual mode of examination, finds a place in all the treatises before us except that of Dr. Andreæ, the first part of which, containing the chapter on the examination of diseased eyes, was published before the observations, which first fixed the attention of surgeons on it, were given to the world.

The catoptrical method has been applied by Dr. Mackenzie to the investigation of the seat of the opaque appearance in glaucoma. We extracted from the Medical Gazette, at the time they were first announced, the results of his observations, which are now recorded in the new edition of his work before us. We think one point has been overlooked, and that is an image reflected from the bottom of the eye. In the healthy state it is scarcely seen, but in the eye, in which the pigment is deficient, it is well marked, and is, we believe, the cause of the peculiar appearance of opacity at the bottom of the eye in glaucoma. It is not a defined image, but being seen through the lens, and not being at the distance behind it corresponding to its focal power, it looks like an incomplete focus. Being the reflection from a concave surface, it moves in the opposite direction to the light. In fungous tumours at the bottom of the eye in the commencement, the reflection differs only in colour and brilliancy from what is seen in glaucoma; but as the tumour advances forward, the reflected image is thrown forward in a corresponding degree, and hence the appearance is as if the whole interior of the eye were illuminated. We think that in glaucoma the state of the lens only modifies the colour of the reflection from the bottom of the eye; and we do not believe, as Dr. Mackenzie now supposes, that the appearance of opacity at the bottom of the eye, peculiar to glaucoma, is a lenticular image at all.

Professor Chelius thinks that the whitish yellow point at the bottom of the eye in glaucoma, and when the black pigment is deficient, is the entrance of the optic nerve; but he does not reflect that if it were so, the bright point ought always to be distinctly seen, as the entrance of the optic nerve is not covered by pigment. The whitish yellow point we consider, as has been said above, a reflection from the bottom of the eye, and the reason it is almost always seen at the inner and lower side of the middle of the bottom of the eye—the region of the entrance of the optic nerve—is that in examining an eye the light is always allowed to fall from above and from the temporal side, consequently, the image being formed by reflection from a concave surface, is on the side opposite to the light. If this explanation of ours be correct, the account given by Professor Chelius is a curious illustration how an erroneous opinion may be apparently well supported by indisputable facts.

M. Carron du Villards remarks, (vol. ii., p. 542,) in speaking of gonorrhœal ophthalmia, "The inoculated disease almost always affects but one eye, and I have observed that in left-handed individuals it was the left eye which was effected, and *vice versâ*."

Dr. Mackenzie gives a section on Artificial Ophthalmiæ, which illustrates all the importance of an accurate ophthalmoscopia.

"When a suspicion arises that a number of soldiers together are simulating puro-mucous ophthalmia, or endeavouring to produce serious injury of their eyes by the use of irritants, the suspicion will of course be increased, if the disease is almost exclusively confined to the privates or non-commissioned officers of a regiment, without affecting the commissioned officers or the women and children; also by the circumstance of the inflammation being very frequently confined to one eye, and that almost always the right." (*Mackenzie*, p. 522.)

After discussing ophthalmoscopia for real diseases, M. Carron du Villards adds: "But medical jurists, and especially those attached to recruiting parties, are called upon to examine diseases which are often simulated. These are principally myopia, amaurosis, opacities of the cornea, chronic ophthalmia with loss of the cilia." (Vol. i., p. 219.) At p. 222, the same author tells us that he knows a man, unworthy of the name of a physician, who has made an enormous fortune by producing, artificially, specks of the cornea in young persons liable to be drawn into the military service.

On the subject of erysipelas of the eyelids, Dr. Mackenzie relates, from Dr. Piorry, a case which, with numerous others of a similar character recorded by the same physician, makes it appear that one of the modes, perhaps the most frequent but least suspected mode, in which erysipelas of the face or scalp proves fatal, is the spreading of the inflammation from the eyelids to the cellular tissue of the orbit, and the termination of it in abscess within that cavity. "It sometimes happens," he adds, "that the cellular membrane of the orbit, although considerably affected, does not suppurate. On the subsiding of the acute symptoms, the eyeball in such cases is found to be deprived of its power of motion, is protruded, or has even become amaurotic from the pressure it has undergone." (p. 122.)

As local treatment for erysipelatous inflammation of the eyelids, M. Carron du Villards, after mentioning mercurial frictions and the two English methods by fine punctures and by cauterization with the nitrate of silver, says: "Professor Rasori treated erysipelas of the face, and especially of the eyelids, with compresses soaked in a cold concentrated solution of tartar emetic (ʒj.-oi.) This has always succeeded with me, and is what I employ by preference." (p. 237.) M. Velpeau has found, in his practice at La Charité, the plan by fine punctures or scarifications

4287

of the skin the most successful in erysipelas of the eyelids. In making the scarifications and in opening abscesses of the eyelids, MM. Velpeau and Carron du Villards recommend great caution, lest the eyeball should be wounded. The latter mentions his having been three times witness of such an accident. One of the cases was in the person of an unfortunate physician of Malesherbes, M. Benod, who had both his eyeballs evacuated in the attempt of an imprudent *confrere* to open erysipelatous abscesses in the lower eyelids. (Vol. i., p. 238.)

Dr. Mackenzie gives a section on Malignant Pustule, as the eyelids are a very frequent seat of it. M. Carron du Villards treats of the disease as it occurs in the eyelids at considerable length, and that from personal observation. In the work of M. Vidal (de Cassis), considerations of high practical importance on the disease in general will be found. Fortunately, malignant pustule is not much known in this country. "I am not acquainted," says Dr. Mackenzie, "with any account of this disease, as observed in Great Britain, unless the cases published under the name of *glanders in the human subject* are to be regarded as instances of malignant pustule, to which it cannot be denied they bear in many respects a striking similarity." (p. 127.) Dr. Andreæ, from multiplied experience, believes that, though malignant pustule is most frequently produced by animal contagion, it may sometimes occur without it. (Part second, p. 49.)

The method of destroying small nævi materni by vaccination was first described in this country, in 1827, by Mr. Hodgson and Mr. Middlemore, of Birmingham. M. C. du Villards, in the work before us, claims having employed vaccination for the cure of nævus in 1822. "In a memoir presented to the Minister of the Interior in 1812, and for which my father was awarded one of the great prizes instituted by Napoleon for the encouragement of vaccination, it was stated that inoculation with the vaccine virus effected the resolution of a great number of indolent glandular and other tumours.........In 1822 I was consulted about an infant six weeks old, which had on the right eyebrow a nævus of the size of a lentil. I inserted some of the vaccine virus into this tumour by means of a small grooved needle. The same thing was done on both sides of the tumour. Three pustules soon appeared, and after running through their stages, left the small congenital tumour completely obliterated." (Vol. i., p. 357.)

An operation has been performed by Mr. Hunt, of Manchester, in a case of traumatic ptosis, which consisted in removing a transverse fold of integument from the eyelid, of such an extent and from such a place, that when the edges of the wound became united, the eyelid was attached to that portion of the skin of the eyebrow upon which the occipito-frontalis acts; by which means the action of this muscle was substituted for that of the levator palpebræ lost. It has been suggested that Mr. Hunt's operation might be tried in cases of paralytic ptosis, when no signs of improvement from treatment appear. It is true that in paralytic ptosis the epicranius muscle is active, its power depending on the nervous stimulus of the facial nerve; but it is to be remembered that, besides the levator palpebræ, the other muscles of the eye supplied by the third pair are generally affected; hence, as M. Rognetta very justly remarks (p. 445), such an operation in paralytic ptosis would not only be useless but even injurious, for the patient would continue to squint and have double vision. However, if there was incurable paralytic ptosis on both sides, the operation might be performed with advantage on one side.

Of all the diseases affecting the eyelids, ectropium is that which has perhaps exercised the operative ingenuity of surgeons most. This is not to be wondered at, considering the various causes giving rise to eversion of the eyelids, and considering the great deformity which this state produces and the injury which always accrues in a greater or less degree to the eyeball. In an article on plastic surgery in a former Number, we had occasion to consider certain very ingenious operations for ectropium. Dr. Mackenzie relates a case, first published some years ago, illustrative of a blepharo-plastic operation which Mr. Wharton Jones has found successful in eversion and shortening of the upper eyelid, from contraction of the skin consequent to burns. The peculiarity of the plan consists in the two following points: 1st. The eyelid is set free by incisions, made in such a way, that, when the eyelid is brought back into its natural position, the gap which is left may be filled by approximating its edges, and thus obtaining immediate union. Unlike the Celsian operation, the narrower the cicatrice the more secure the result. 2d. The flap of skin embraced by the incisions is not separated from the subjacent bone, but advantage being taken of the looseness of the cellular tissue between the skin and the bone, the flap is pressed downwards, and thus the eyelid is set free. This method of Jones is described by Velpeau (pp. 105-13), and Vidal de Cassis (pp. 530-1) in the works before us, as one of the simplest and best of the blepharo-plastic operations. It has been performed by M. A. Berard without success, and by M. Velpeau, at La Charité, successfully in one case, unsuccessfully in another, in which erysipelas came on and marred the result.

Before leaving the subject of ectropium, we would make a remark on the literature of an operation proposed and practised by Professor Jaeger of Vienna, the object of which is to increase the perpendicular length of the eyelid, as well as to reduce its transverse elongation. The operation is mentioned in Juengken's Manual of the Diseases of the Eye, and particularly described in an inaugural dissertation by Dreyer, published in Vienna in 1831, under the eye of Professor Jaeger himself.* Mr. Lawrence in his treatise on the Diseases of the Eye (p. 350), describes the operation in question from Juengken's Manual, and in the next page has a note in regard to the same operation as described by Dreyer, but which he supposes another and a different one of Jaeger's, and which he says he does not understand. Mr. Middlemore, misunderstanding the description of the operation given by Mr. Lawrence from Juengken, remarks in a note to his account of the ordinary Celsian operation (p. 787), "Mr. Lawrence attributes this practice to Jaeger and Juengken, but it was advised, practised, and very circumstantially described a hundred years ago by Platner, as well as the proper bandages and other means required for keeping apart the edges of the wound." But the mistake regarding Jaeger's operation, among certain of our English writers, does not end here.—Mr. Samuel Cooper, in the

* There is a very good account of the operation in the Medical Gazette, vol. xvii., p. 721, 1836, by Dr. W. Brown, of Glasgow; and a critique on it by Mr. Wharton Jones, in vol. xviii., p. 224 of the same Journal.

last edition of his Dictionary, describes the operation correctly enough, nevertheless, quotes, in reference to it, the remarks of Mr. Middlemore, made, as we have seen, under a misapprehension of the nature of the operation.

In the ordinary operation for entropium, the fold of skin removed, is parallel to the edge of the eyelid. A mode of operating practised by M. Janson, of Lyons, consists in the excision of a vertical fold of skin extending to near the free edge of the eyelid. "This method, ingenious as it is easy," it is said, "has constantly succeeded in the hands of its author." (Carron du Villards, vol. i., p. 327.) Dzondi followed up the ordinary excision of a horizontal fold by that of a vertical one. In a case "excessivement grave," M. Segon obtained "une guérison complète" by following up the excision of a vertical fold by that of a transverse fold in the whole length of the eyelid.

M. Carron du Villards has performed a modification of Janson's operation in one case with advantage. It consisted in making in the margin of the eyelid, five or six excisions similar to those prescribed by Janson, with the only difference, that they were less deep. By the subsequent cicatrization, an inclination outwards was given to the edge of the eyelid.

Professor Chelius mentions an operation for entropium, by Brach,* which does not, like Crampton's, involve the whole thickness of the eyelid. Though apparently more simple than Crampton's, it will be found to require in the performance of it more delicate manipulation. M. Velpeau confounds Brach's operation with that we have already mentioned, as having been performed by Mr. Hunt for traumatic ptosis. The two operations certainly agree in this, that the bit of skin removed is at a distance from the free edge of the eyelid, but this is done with different views.

Dr. Mackenzie mentions a plan of operating in cases of chronic inversion of the lower eyelid, which Mr. Wharton Jones has performed with perfect success. "Having made an incision through the whole thickness of the lid, perpendicular to its edge, near the outer canthus, he cuts out a piece of the skin, and then fixes the lid in the everted position, as in Sir Philip Crampton's operation." (*Practical Treatise*, p. 215.)

M. Rognetta mentions (p. 445), a case of congenital *mediate* anchyloblepharon. There was a small hole at the external angle, by which the tears escaped.

The lower portion of the lachrymal gland, as it appears to us, has been strangely overlooked by surgeons, because in most books, in that of Chelius before us for instance (p. 10), we find the lachrymal gland spoken of as if it were all contained in the lachrymal fossa, and therefore not liable to be wounded. Dr. Mackenzie, however, refers to the exposed situation of the inferior portion of the lachrymal gland, or glandulæ congregatæ, and of the excretory ducts of the gland. In fact, the outer part of the upper eyelid cannot be deeply wounded without the parts just named being implicated.

M. Velpeau mentions the circumstance of a M. Nelle, who having left the lachrymal gland in the orbit after the extirpation of the eye, was

* This was previously described in Preuss. Medic. Vereins-Zeitung, 1837, No. vi. p. 27; and Zeis, Handbuch der plastischen Chirurgie, p. 392.

forced six months after to remove it also in consequence of the abundant lachrymation it kept up.

Professor Chelius gives (p. 37) a chapter on obliteration of the excretory ducts of the lachrymal gland. But such a condition of the lachrymal ducts is merely assumed to account for the dry state of the eye. It is not established by any direct unequivocal observation—which it would be next to impossible to make on the living eye, and even in the dead eye, we all know how often persons are unsuccessful in demonstrating the ducts of the lachrymal gland. Moreover, supposing the ducts obliterated, that would not be the sole condition of the disease, for the gland itself would be the part principally at fault. Though the pathology of the lachrymal organs has been very fully and ably investigated, it appears to us, that several obscurities and crudities still hang about the subject, which we can expect to give way only before increased physiological knowledge.

The laying open of the lachrymal sac from within the lower eyelid, proposed by Pouteau, (Mélanges de Chirurgie, Lyon, 1760, p. 100,) has lately been recommended as new in this country. A. Petit, Pellier, Leveillé, and Bouchet were the only persons in France who considered the procedure worthy of their attention. It is a method, we think, which should not be adopted. The slight cicatrice which an incision of the skin may leave is not to be weighed with the morbid change of the conjunctiva which is likely to result from the constant contact of the morbid secretions from the lachrymal sac.

M. Vidal de Cassis, in discussing the advantages and disadvantages of the insertion of a tube into the nasal duct, dwells at considerable length (p. 557) upon a question first raised, we believe, by Dr. Mackenzie, viz., "whether the tears actually flow through the metallic canal furnished to them by this contrivance, or descend merely on the outside of the tube, as they do along the surface of a style." M. Vidal without appearing to be aware of the opinion previously expressed by Dr. Mackenzie, maintains with this practitioner the latter opinion. "The tube," says Dr. Mackenzie (p. 258,) "probably operates more in dilating the duct than in affording a channel for the tears; and I am disposed to think that a gold style furnished with a round head, of such thickness as to allow it to sit easily in the lachrymal sac, but to prevent it from sinking down the duct, and over which the sac and the skin should be healed, and which should be worn for life, might answer the purpose just as well as a tube, or better." (*Practical Treatise*, p. 258.)

Though the insertion of the tube is for the most part attended by immediate and very striking relief of all the symptoms, it is not to be forgotten, that it not unfrequently becomes necessary to remove it again ere long, if it has not of itself fallen out.—"In seven years," says M. Carron du Villard, "I have extracted twenty-five tubes; and I know a great number of persons who have been operated on by others. In one week, three persons presented themselves to Professor Cloquet at the clinic of the "Ecole" to have their tubes taken out." (Vol. i., p. 449.) In explanation of the great number of cases in which tubes have been inserted into the nasal duct and afterwards requiring to be extracted, which the above remarks would lead us to infer, it is to be remembered how indiscriminately and prodigally the operation was had recourse to some years ago in France, where it was revived by Dupuytren. M. Carron du Villards, makes the following observations on the practice:

"Led away by the facility and brilliancy of this method, most French surgeons of the day adopted it. Their example was followed by a great number of foreigners. Attracted by the noise of Dupuytren's wonderful cures, patients flocked to the Hôtel Dieu. Whatever was the nature of the case—whether disease of the lachrymal sac or nasal duct,—the inexorable tube was inserted. From this infatuation there resulted many mistakes, and it was soon found out that the use of the tube was not unattended by inconveniences nor accidents, nor even relapses. It could not be otherwise; for Dupuytren, after having opened the sac, thrust in the tube without previously making any examination of the state of the nasal duct." (Vol. i., p. 446.)

Before passing to the consideration of the diseases of the eyeball, we would refer to a curious case of ophthalmoptosis related by Dr. Mackenzie (p. 280), which has much analogy with one mentioned in the Pathology of Verduc, (vol ii., p. 44,) and cited by M. Jeanselme, (p. 496.) We would also refer to a case of ligature of the right common carotid by M. Velpeau for aneurism by anastomosis, occurring in both orbits after a blow on the back of the neck, (p. 486.)

As most of the Diseases of the Eye are either inflammation itself or the effects of it, and, as all our operations on the eye must be regulated by the kind and degree of inflammation we expect to follow, the study of the ophthalmiæ must ever be considered the master-key of the subject. Considering also that there enter into the structure of the eye tissues of all kinds, and knowing that inflammation, simply considered, of a tissue is much the same in whatever part of the body it exists, the study of the ophthalmiæ is well calculated to assist that of inflammation in general. A correct diagnosis of the ophthalmiæ is of paramount importance. It may truly be said, that more eyes are lost in consequence of a wrong diagnosis than from ill-directed treatment when the diagnosis has been correct. The young surgeon ought therefore to embrace every opportunity to examine inflammations of the eye, so as to become familiar with the various forms they put on. It is not the possession of *recipes* for salves and eye-waters that is of much consequence, but it is to be able to distinguish under what circumstances they should be employed. The various applications to the eye which have been recommended, are all very good and useful, but they must be employed with method and discrimination.

What requires to be principally noticed in regard to M. Velpeau is the great *fracas* he makes about his views regarding the inflammations of the eye. He tells us that there are no such things as catarrhal, scrofulous, rheumatic, &c. ophthalmiæ. If we turn to the work of Mackenzie, we shall find that the epithets catarrhal, scrofulous, rheumatic, arthritic, are conventionally employed to designate certain well-marked forms of inflammation of the eye, without its being meant that they are always a manifestation of the general disease, the existence of which they would seem to indicate. We believe that this is pretty well understood, at least in this country: we believe it is also admitted that, when we speak of conjunctivitis, sclerotitis, corneitis, and the like, it is understood that the inflammation is not confined to the particular texture indicated by the name: in short, that the nomenclature of the diseases of the eye—

the dynamic diseases especially—is, as in other departments of nosology, necessarily to some extent conventional, always requiring particular descriptions and definitions. It is, therefore, somewhat remarkable to find M. Velpeau—the whole tenor of whose statements (as contained in the work before us, edited by M. Jeanselme,) indicates an origin from a person who has but just turned his attention to the subject, and to whom consequently everything is *new*, and therefore everything a *discovery*;—it is somewhat remarkable, we say, to find M. Velpeau stepping forward and proclaiming that the current opinions in regard to the inflammatory affections of the eye are all founded in error! And the way M. Velpeau proves this is by giving names of his own to certain well-known forms of inflammation, and then turning round and saying that what surgeons call catarrhal ophthalmia for instance does not exist, or is at the most nothing but the form of inflammation now for the first time correctly described and designated by him! But he does not always do so much. In many instances he separates what is naturally connected, and confounds what is essentially different. As an example of the way M. Velpeau corrects the errors of former authors, we may refer to what he says (p. 31) of granular conjunctiva, which he designates "blephorite granuleuse," and tells us that it has not been described apart by authors. Now it has been described in all sorts of ways—sometimes as the consequence, sometimes as the harbinger of purulent ophthalmia, and moreover been the subject of fierce contention. Again (p. 284) we are told that the therapeutics of iritis is but little advanced! In regard to such statements, we are at a loss to say whether the ignorance or boldness displayed be the more remarkable. M. Velpeau frequently alludes to the works published in this country on the diseases of the eye; but in general only to evince by his misstatements how little familiar he is with them. Thus we do not know where he could have found it stated, as he has it at page 211, that Lawrence and Mackenzie order 15 to 20 and 30 grains of tartar emetic in the twenty-four hours in cases of acute corneitis, and that they sometimes combine it with opium and sulphur. The use of calomel in iritis is discussed at page 287 under the head of "Purgatives." To conclude this notice of M. Velpeau's views regarding the ophthalmiæ, it is most provoking the manner in which he presumes to criticise that of which he has not taken proper pains to inform himself, and all this without having made any useful contribution, so far as we can see. No doubt a person but little familiar with the subject might, on reading M. Velpeau's lectures, be led to suppose that he is the sole discoverer of many of the remedies most effectual in combating the ophthalmiæ and of the indications for their employment.

Mr. Morgan, in the work we lately had occasion to notice, goes as far one way as M. Velpeau does the other, when he lays so much stress on the inflammatory diseases of the eye being nothing but catarrh, rheumatism, scrofula, &c.

The inflammations of the conjunctiva, from their frequency and in some cases their extraordinarily destructive nature, demand the surgeon's most serious consideration. It is in the puro-mucous inflammations of the conjunctiva that local applications of an astringent and a stimulating nature produce their most striking effects; but it is to be remarked that, though the injection of the conjunctiva covering the eyeball be frequently removed as if by enchantment, it will be found, on examining the state of the palpebral portions of the conjunctiva, that the cure is only half effected. This, however, is a great deal; and it would be well if we could always calculate on doing as much: but, unfortunately, cases occur in which Dupuytren's insufflations of calomel, Guthrie's black ointment, or any other boasted remedy of the kind is of little avail. Nor is general antiphlogistic treatment alone successful. "A treatment," says Mackenzie, "partly antiphlogistic or soothing and partly stimulating, is the most successful in the cure of all the puro-mucous ophthalmiæ." (p. 371.) In the severer forms of puro-mucous ophthalmia accompanied by chemosis, all the ordinary plans of treatment having been too often found ineffectual in shielding the eye from destruction, M. Sanson has fallen upon the idea that, by extirpating the conjunctiva oculi, he should strike at the root of the evil by removing its seat! To say nothing of the ulterior effects of such an encroachment on the organic constitution of the eye, it is to be remarked that the destruction of the eye in such cases is caused not altogether by the excessive inflammatory action of the conjunctiva, but by the injury accruing to the cornea from the pressure exerted on it by the chemosis: a fact pointed out by Dr. Mackenzie in all the three editions of his work, the first of which bears date 1830; and also dwelt on at considerable length by Mr. Middlemore in his treatise published in 1835, as well as in lectures which appeared in the Medical Gazette in November, 1832, et seq. All that can be effected by surgery, therefore, may be done in a milder and less objectionable manner. Scarification of the conjunctiva, the incisions being made concentric with the cornea or in no particular direction, is what has been ordinarily employed; but Mr. Tyrrell has recently recommended the incisions in the conjunctiva to be made in a direction radiating from the cornea and at the places corresponding to the intervals between the insertion of the recti muscles. We have, in a former Number, adverted to Mr. Tyrrell's paper on the subject: here we would only remark that, without offering the slightest objection to the practice, we cannot agree in the rationale of it. M. Sanson's practice of extirpating the ocular conjunctiva is a complete refutation of it in the gross; and Mr. Wharton Jones's remarks, published some time ago in the Medical Gazette, are a refutation of it in detail. M. Velpeau tells us he has derived great advantage from the direct application of leeches to the ocular conjunctiva in inflammatory chemosis.

M. Carron du Villards and also M. Jeanselme give a chapter on Miasmatic Ophthalmia—"la mitte ou ophthalmie méphitique" of scavengers and nightmen, which used to be common in Paris. Dr. Furnari, who has studied with great care the diseases of the eye among the different professions, has made a great many observations on this subject, and it is to him that both Carron du Villards and Jeanselme owe their information. The ophthalmia in question appears to be of a puro-mucous character.

An opinion, enunciated some years ago by Dr. G. Gregory, that smallpox pustules do not form on the conjunctiva and cornea, and maintained in France by M. Gersent, has been lately well supported and illustrated by Mr. Marson, Surgeon to the Smallpox Hospital. Mr. Morgan, in his zeal to prove the analogy subsisting between the diseases of the eye and those of other parts of the body, says, in reference to variolous ophthalmia, "No one can doubt for a moment that this is a true pustular disease." (p. 34.) Dr. Gregory and Mr. Marson's observations above alluded to are opposed to Mr. Morgan's assertion, that variolous eruptions may occur either on the conjunctiva of the eyelids only, or they may extend generally over the whole surface of that membrane. In fact, the eye suffers in smallpox from common inflammation merely, though of a very severe form. It is an onyx not a pustule that leads to the destruction of the cornea. The fact of the onyx not appearing till the eruption of the general disease is on the decline proves, according to Dr. Gregory and Mr. Marson, that it is not a primary or essential feature of the disease. We would add that the onyx occurring in the substance of the cornea—a tissue quite different from that of the skin—cannot be considered of the same nature as a pustule of the skin, though it resembles it in appearance. Considering the anatomical circumstances of the conjunctiva, if it be affected in any way at all analogous with the skin in smallpox, the external characters of the eruption, as M. Vidal de Cassis remarks (p. 260), must be very different from those presented by that of the skin.

In his article on phlebitic ophthalmia, Dr. Mackenzie gives a series of cases illustrating its rise from,—1st, Inflammation of a distant vein, produced by a wound, or by tying the vein ; 2d, Suppurative inflammation of the uterine branches of the hypogastric veins in puerperal women ; 3d, Phlebitis occurring in erysipelas, or diffuse cellular inflammation ; 4th, Phlebitis arising in the course of febrile diseases.

In illustration of phlebitic ophthalmia from suppurative inflammation of the hypogastric veins in puerperal women, Dr. Mackenzie quotes a case, observed by Dr. Graves, of Dublin, of "fatal phlegmasia dolens after parturition, in which one of the eyes was affected." This case is also quoted by M. Rognetta, under the head of "Phlegmasia alba dolens de la conjonctive." M. R. says, in regard to phlegmasia dolens generally, "It is now agreed that the nature of this malady is purely nervous"! As M. Rognetta concludes his article with the following remark, "The affection being as yet known only by this single case [?] of Professor Graves, I can say no more on the subject," he cannot have confounded the affection of the eye under consideration with the disorganizing inflammation of the eye, which has been observed to take place in consequence of injury or disease of the fifth pair.

The subject of sympathetic action, whether of a healthy or morbid character, is one of very great importance in medicine. In the healthy state the most marked sympathy prevails between the two eyes, as far as regards their visible motions. Nor is it less striking in regard to those actions of which each individual must judge for himself, and that only by results—witness the one visual perception from two dissimilar retinal affections. In many circumstances the two retinæ act but as one. If such close sympathy exist between the eyes in health, it is not surprising that we should in some instances find the one taking on a similar diseased action with the other.

In commencing his account of sympathetic ophthalmia, Dr. Mackenzie observes (p. 523), "The general nature of the sympathetic affection, which I am about to illustrate, is inflammation, not of the iris alone, but involving the whole of the internal textures of the eyeball, especially the crystalline and vitreous capsules and the retina; coming on generally in five or six weeks after an injury of the opposite eye, and terminating most frequently in atrophy and total amaurosis of the eye secondarily affected. The one also which received the original injury generally ends, or has already ended, in amaurosis and softening of the globe."

Sympathetic ophthalmia has hitherto proved so little amenable to treatment, that the total and timely destruction of the injured eye has been proposed; but such a proceeding, it is obvious, can only be justified by extreme circumstances, and after the case has been subjected to the nicest discrimination. First suggested by Mr. Wardrop, from the knowledge of a similar practice being successful in veterinary surgery, the destruction of the injured eye has been actually adopted, though with a somewhat different view, by Mr. Barton, of Manchester. The principle of the proceeding, as suggested by Mr. Wardrop, we think just; for as the retina of the injured eye is evidently one of the principal parts affected, and as the retinæ are constantly working together, disease of one will be a constant source of irritation to the other.

Dr. Mackenzie gives a chapter on Intermittent Ophthalmia. In the former edition of his work he expressed a doubt as to the existence of any disease of the eye, so regularly periodic in its accession, as to warrant the appellation of intermittent ophthalmia. He, however, now gives a case which he says has induced him to change his opinion. The case was that of a gentleman, aged twenty-four, who had been troubled with scrofulous ophthalmia up to the age of ten. For ten months before he consulted Dr. M., the conjunctivæ had been affected with considerable redness, and this symptom presented exacerbations of a distinctly periodic character. The attacks came to a crisis in about thirty-six hours from their commencement, the redness after this gradually decreasing, till the eyes recovered something like their proper colour, and the whole process occupying generally six or seven days. The last time Dr. M. saw the patient was about four years after the commencement of the complaint, which still continued with little change—all the remedies of the most eminent surgeons and oculists of these countries having proved fruitless.

The following observation of M. Rognetta may or may not have something in common with the above kind of affection: "There are," says he (p. 130), "cases of chronic conjunctivitis very slight in appearance, which are connected with slow affections of the meninges of the brain." He then mentions the case of a girl who was "affected with a *conjunctivite intercurrente* of one eye, simulating the catarrho-scrofulous form. Each return of the injection of the eye was accompanied by a certain unusual irritability, headach, and photophobia, not at all in proportion to the slightness of the ophthalmia." Encephalic symptoms afterwards appeared, and death supervened on the sixth or seventh day.

Here would be the place to introduce the Monography of Dr. Eble; but as this article has already extended to so great a length, and as we have had occasion in a previous Number to notice his views regarding the Egyptian ophthalmia, we forego any particular examination of it. We must, however, give some analysis of Dr. Von Ammon's prize essay on Iritis.

We have already noticed some points in chapter first. Chapter second is on the nature and treatment of iritis in general. Traumatic iritis is considered in the third chapter. In the fourth the author treats of "iritis serosa," or inflammation of the serous lining of the anterior chamber, of which he describes a rheumatic, a scrofulous, and a mixed-cachectic species. The fifth chapter is entitled, "De Iritide parenchymatosa et de variis ejus formis;" and as forms of parenchymatous iritis we find admitted, "Iritis arthritica," "Iritis syphilitica," "Iritis scrophuloso-psorica," and "Iritis scrophuloso-plicosa." In the sixth chapter the author endeavours to establish the nosology, diagnosis, and treatment of what he calls "*Iridoperiphakitis!* vel uveitis vel iritis serosa posterior vel inflammatio iridis et capsulæ lentis."

In the above divisions and subdivisions of iritis, it must be confessed, there is too much wire-drawing and conceit.

In speaking of traumatic iritis of one eye, Dr. Von Ammon alludes to the inflammation which is apt to supervene in the other, and thinks to ward it off by applying leeches, not near the eye, but on the temples or behind the ears, and to the eye itself cloths soaked in cold water containing belladonna in the proportion of one grain of the extract to the ounce.

The author tells us he has had several times an opportunity of anatomically examining the iris after gouty inflammation, and has found its natural texture exchanged for an amorphous condensed cellular mass, in which separate vessels and nerves could no longer be recognized.

Those forms of gouty iritis in which, amidst severe gouty pain in the head, vision is often wholly lost in a single night, and in which there is dilatation of the pupil and a glaucomatous appearance, which he calls opacity of the vitreous body, the author considers rather as secondary, arising from inflammation of the choroid and vitreous body. This is in the main what is now generally held by authors. Thus we find that Dr. Mackenzie, in considering the "changes in the iris and pupil, glaucoma, amaurosis, atrophy of the eye," under the head of "Arthritic Iritis," remarks:

"The form of disease just described, although accounted by Beer one of the varieties of arthritic iritis, evidently involves the other textures of the eye more than the iris. Mr. Lawrence and Dr. Canstatt have therefore objected to Beer's classification of the affection, the former describing it under the name of *arthritic inflammation of the internal tunics*, and the latter under that of *choroideitis*. From the suddenness with which vision is extinguished in the affection, along with the dilatation of the pupil which attends it, it is probable the retina is as much the original seat of the disease as any other texture of the eye, and certainly more so than the iris. Mr. Tyrrell describes the same disease under the name of *retinitis*." (p. 484).

We consider it a *choroido-retinitis*, extending on the one hand to the iris, and on the other leading to changes in the vitreous and crystalline bodies, besides destroying the membrane of the pigment; on the loss of which, as we have already mentioned, the appearance of opacity in the vitreous body depends.

As a specimen of our author's therapeutics we give the following: Of collyria he says, "Collyria in universum, in iritide, plus damni quam commodi ferunt." He recommends the inunction of calomel and opium around the eye, and employs belladonna in bags hung over the eye; for this purpose the powder of the dried herb is mixed with powdered sweet almonds in the proportion of one drachm of the former to one ounce of the latter.

At the commencement of iritis serosa he employs simply antiphlogistics and cooling laxatives, and has recourse to *specifics* only when the cure does not go on. What he means by specifics are anti-rheumatics in the rheumatic form of iritis serosa, and anti-scrofulous remedies in the scrofulous form. He considers the muriate of baryta with cicuta a powerful anti-scrofulous remedy, and ascribes to it "miram vim in membranas bulbi serosas;" and remarks, that though it does not cure quickly it cures safely. In the rheumatic form of iritis serosa he praises tartar emetic, which he says acts here as powerfully as in pleuritis.

In syphilitic iritis he advises the corrosive sublimate dissolved in sulphuric ether; but he characterizes syphilitic iritis as "morbus curatu difficilis."

As the treatment of iritis is pretty well understood in this country, we do not think it necessary to make any comment on the above.

The figures, eighteen in number, are coloured representations in large of the iris and pupil simply, not of the entire eye.

We now pass on to the consideration of a few of the more interesting points connected with the organic changes of the eye consequent to the ophthalmiæ. And first a word on *pannus.* This morbid condition of the conjunctival layer of the cornea, in general so rebellious to treatment, Professor Jaeger tells us he has sometimes succeeded in removing, by exciting in the eye a new attack of blenorrheal ophthalmia, by means of inoculation, and then, by well-directed treatment, bringing the inflammation to a favorable termination. This, it is obvious, is a hit or miss proceeding, even if we could always calculate on the cornea becoming clear in the cases, in which we might succeed in saving the eye from total destruction.

Conjunctival xeroma, or xerophthalmia, has of late attracted considerable attention in this country, in Germany, and also in France. It is, says Dr. Mackenzie, "a very peculiar state of the conjunctiva, the result of long-continued and ill-treated inflammation of that membrane. It has been described by Mr. Travers under the name of *cuticular conjunctiva.* He mentions that he had seen cases of this conversion of the conjunctiva into a rugous and opaque skin, go the length of knitting the lids close to the globe, and obliterating the sinus palpebrales." This disease is supposed by some to have for its proximate cause obliteration of the lachrymal ducts. On this point we would refer to what we have already said regarding obliteration of the lachrymal ducts, and remark that, supposing such a condition does exist in xerophthalmia, it ought rather to be looked upon as an effect than as a cause. M. Rognetta thinks it is owing to an affection of the fifth pair, producing a deranged state of the innervation of the part, he, therefore, discusses it under the head of "Neuroses conjonctivales." M. Vidal de Cassis entertains a similar opinion. The view of the matter given by Mackenzie, we believe, is the obvious and rational one, viz., that the disease consists in a change in the structure of the conjunctiva, in consequence of long-continued inflammation, accompanied by a corresponding change in the natural action of the part. Like other changes in the intimate structure of organs, conjunctival xeroma has been found incapable of radical cure.

M. Sanson, either overrating the reproductive powers of the tissues, or supposing that a conjunctiva is not of very essential importance to the eye, has adopted here the practice we have already mentioned, as having been employed by him in purulent ophthalmia, viz., excision of the conjunctiva, the seat of the disease! but without any good result. "With the intention," says Dr. Andreæ, (Part ii., p. 411,) "of producing a sloughing and afterwards an exfoliation of the corneal conjunctiva, I cut away the dry conjunctiva all round the cornea; but this also was of no service."

Before examining the views regarding the formation of staphyloma, so-called, of the cornea and iris, contained in the works before us, we may notice a singular operation performed by M. Lisfranc, in a case of total staphyloma, but not of large size, in a young man, who was received into the hospital of La Pitié in 1836—the intentional formation, namely, of an anchyloblepharon. "M. Lisfranc, in order to conceal the deformity, and to oppose the farther progress of the disease, united the two eyelids together by suture, after having of course rendered the edges raw; but there necessarily resulted another disagreeable deformity—the anchyloblepharon. This singular operation, besides leaving the wolf in the sheep-fold," remarks Dr. Rognetta, "and keeping us ignorant of what might afterwards become of the tumour, does away, it is evident, with the immense advantage of applying an artificial eye. Moreover, it is to be asked, is the operation of uniting the edges of the eyelids not more painful and more tedious than excision of the cornea?" (p. 237.)

A peculiar change in the structure of the iris, occurring sometimes as a consequence of chronic, neglected, or ill-treated iritis, has been described by Professor Jaeger, of Vienna, under the name of *staphyloma iridis*, and more recently by Dr. Klemmer, under that of *iridoncus* or *iridoncosis*. Dr. Mackenzie describes it in the first paragraph of his section on Staphyloma, under the title of *staphyloma uveæ* or *iridoncosis*. Jaeger supposes the change to consist in an attenuated state of the substance of the iris and a protrusion of the uvea through it. Klemmer, on the contrary, contends that it is not a thinning but a thickening of the iris; not a shining through of the uvea, but a deposition of coagulable lymph in the parenchyma of the iris. Klemmer relates only one dissection, and that not of the human eye, but of the ox.

In regard to the formation of staphyloma of the cornea and iris, Dr. Mackenzie, adopting the views of Mr. Wharton Jones, says that it is not owing to adhesion of the iris to the cornea, as generally taught, and as we find laid down in all the other works before us; but the cornea having been first more or less destroyed, it is a covering up of the exposed iris by a new substance altogether, which lays the foundation for staphyloma, and constitutes an essential character of the disease. (p. 569.) We believe that adhesion between the iris and cornea, independent of previous penetrating ulceration, does not take place.

Of course, when an inflammation of the eye has run so disastrous a course as that the conditions for the formation of a staphyloma are laid, any prophylactic treatment which may be adopted can have for its object, not to save the eye as an organ of vision, but to prevent it from becoming a tumour, which not only causes great deformity, but is a source of considerable irritation to the opposite eye as well as in itself—so much so, that the patient seeks for its removal by operation sooner or later. Led by the view he takes of the mode of origin of staphyloma, Mr. Wharton Jones has adopted a peculiar preventive treatment, for a full explanation of which we must refer to Dr. Mackenzie's work. (p. 573.) Mr. Wharton Jones, in the paper in the Medical Gazette, in which he fully enunciated his views regarding the mode of origin of staphyloma, shows that this at first differs only in degree from prolapsus iridis, therefore, when Chelius speaks, at p. 174, of an old prolapsus iridis never presenting a prominence, he gives a very imperfect and one-sided view of the subject. The fact is, if the prolapsus iridis be within certain limits of size, it gradually contracts and flattens as the inflammation abates and the ulcer closes; but if beyond these limits it never collapses, but becomes covered over with a pseudo-cornea, and a partial staphyloma is the result.

It would be an acquisition of great importance to be able to distinguish non-malignant from malignant tumours in the eye at an early stage. "It may now be regarded," says Dr. Mackenzie, "as a generally received opinion, that frequent instances occur of changes of structure deep in the eyeball, producing all the visible appearances of fungus hæmatodes of the eye, but which do not turn out to be malignant. Such cases are not uncommon after injuries. They constitute a diseased state of the eye, which Beer included along with some other conditions of the deep-seated parts of that organ, under the name of *amaurotic cat's eye.*

"If we extirpate such eyes under the notion that they are affected with fungus hæmatodes, the patient will continue well, and we shall fall into the error of supposing that our operation has been an exception to the general failure which attends the removal of the eye in that disease. This error has probably been committed by Mr. Wishart and by Mr. Porter, in two cases which have been given to the public..... In some of the cases in question, I have observed that the ciliary edge of the iris appears wrinkled, the larger circle is drawn somewhat backwards, while the smaller circle projects forwards, and is broader than usual; the pupil is in a middle state of dilatation, and its edge is fringed with uvea; the surface of the deposition or tumour at the bottom of the eye is of a pale tint and not so defined as in the malignant cases." (*Mackenzie*, p. 606.)

The above observations, so far as they go, are of so much value that we make no apology for the length of the extract.

In Number X. of this Review, we noticed an article on cataract, by M. Maunoir, in the Memoirs of the Medical Society of Observation of Paris; the object of which was to draw, according to the numerical method, some general deductions, in regard chiefly to the success following the operation for cataract. The data were derived from observations made by M. Maunoir at Professor Roux's clinic. In proportion to the imposing effect which figures give to arguments, so ought to be our jealousy in regard to the correctness of the data on which numerical calculations are founded. If correct, we are furnished with a safe and steady stepping-stone to truth—if incorrect, we are confounded by the sudden yielding which takes place, and get involved in a slough of error. In Dr. Ammon's Monatsschrift for May and June, 1839, we find a letter, dated "Paris, 28th Dec., 1838," written by Dr. Schneider to Dr. Fr. Pauli, of Landau, in which we read, amongst other things in regard to the Medical School of Paris, the following remarks on Roux's operations for cataract: "Cataracts are all extracted, whatever be their nature—secondary capsular cataracts—such as adhere to the iris, and in which every other part of the eye is as likely to come out as the cataract itself. He tells his colleagues that he finds extraction not more difficult than phlebotomy, and that he performed it the first time as well as he does now. It always succeeds with him. But the word succeed, in his view of the matter, means the same thing as to finish. He really performs this operation with remarkable celerity, but I have seen him wound the iris very frequently." (pp. 254-5.) If such be M. Roux's practice, we must reject, as inconclusive, whatever M. Maunoir would have us infer from his figures.

It may here be mentioned, on the subject of cataract, that M. Velpeau gives a very good appreciation of the operations of extraction and depression; but at p. 365, it is very absurdly said, in regard to central cataract, that it had not been before described.

The operation of artificial pupil is more or less ably discussed in all the works before us, with the exception of that of M. Rognetta and M. Vidal. The article on artificial pupil, indeed, is perhaps the most imperfect in M. Rognetta's book.

In M. Carron du Villard's book, we read as follows: "Congenital imperforation of the iris is not frequent: there is, however, a certain number of cases on record, the most remarkable of which was observed by Cheselden, for it was it which suggested to him the idea of opening it artificially, and thus to invent a new operation, which made so much noise in the scientific world." (Vol. ii. p. 195.) This same inaccurate story of Cheselden's cases of artificial pupil is repeated by Velpeau and Vidal. The great misconception here displayed seems to have arisen from confounding the congenital cataract case with the two artificial pupil cases related by Cheselden in the same volume of the Philosophical Transactions, viz. vol. xxxv., an. 1728. It is distinctly stated that, in both the cases in which the artificial pupil was made, the closure of the natural aperture had taken place in consequence of the operation of couching. That it is not foreigners only who have misunderstood Cheselden's sufficiently plain though brief account of his first operations for artificial pupil, will be found pointed out, on referring to a note at p. 372 of Dr. Mackenzie's work. In the life of Cheselden, in the Penny Cyclopædia, the same error is committed as that above exposed in Carron du Villards.

We must now bring this article to a close, omitting entirely the subjects of amaurosis and the various states of unnatural and defective vision, as being of too much consequence to be made a tail-piece of. We would remark that, having been compelled to characterize M. Rognetta's article on artificial pupil as the most imperfect in his book, we are happy to say his article on amaurosis is one of the best.

There are still two compositions before us relating to the subject of this article, but which we have not inserted in our list. The one we do not know well how to characterize. It is ostensibly a treatise on the art of preserving the eye in a healthy condition, and of improving the sight; and does contain, in part the second, many good remarks and observations on the subject; but part the first being a sort of romance, we leave it to be handled by the strictly literary reviewers. Speaking as medical reviewers, we should say that Dr. Franz would have been better advised had he published part the second separately, and that in a less inflated style.

As to the other composition, it is enough to say that the author boasts of a secret remedy he became acquainted with in India, which, "strange to say, instead of being applied to the eye, is dropped into the ear!"

Before closing this article, we think it our duty to advert to the unseemly exhibition of the names of certain medical men daily made in the advertisements of two rival Jew spectacle-venders. Even supposing the spectacles thus lauded as good as any other, is it not unjust on the part of these medical men, to say nothing of the dishonour to the profession, to give the weight of their names to indisputable exaggerations, which tend to deceive the public, and indirectly to prove disadvantageous to those really scientific opticians who conduct their business in a way becoming respectable citizens?

---

## Figures and Tables

**Figure f1:**
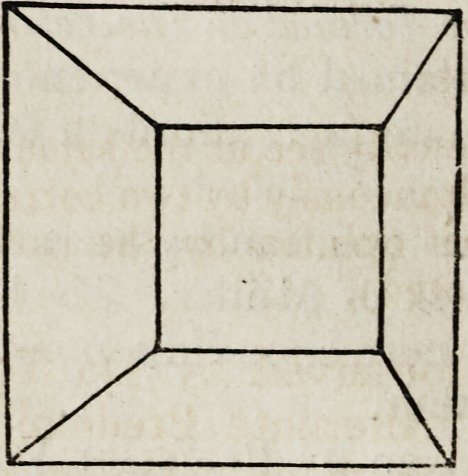


**Figure f2:**